# Biology-Inspired Microphysiological Systems to Advance Patient Benefit and Animal Welfare in Drug Development

**DOI:** 10.14573/altex.2001241

**Published:** 2020-02-28

**Authors:** Uwe Marx, Takafumi Akabane, Tommy B. Andersson, Elizabeth Baker, Mario Beilmann, Sonja Beken, Susanne Brendler-Schwaab, Murat Cirit, Rhiannon David, Eva-Maria Dehne, Isabell Durieux, Lorna Ewart, Suzanne C. Fitzpatrick, Olivier Frey, Florian Fuchs, Linda G. Griffith, Geraldine A. Hamilton, Thomas Hartung, Julia Hoeng, Helena Hogberg, David J. Hughes, Donald E. Ingber, Anita Iskandar, Toshiyuki Kanamori, Hajime Kojima, Jochen Kuehnl, Marcel Leist, Bo Li, Peter Loskill, Donna L. Mendrick, Thomas Neumann, Giorgia Pallocca, Ivan Rusyn, Lena Smirnova, Thomas Steger-Hartmann, Danilo A. Tagle, Alexander Tonevitsky, Sergej Tsyb, Martin Trapecar, Bob van de Water, Janny van den Eijnden-van Raaij, Paul Vulto, Kengo Watanabe, Armin Wolf, Xiaobing Zhou, Adrian Roth

**Affiliations:** 1 TissUse GmbH, Berlin, Germany;; 2 Technische Universitaet Berlin, Berlin, Germany;; 3 Stem Cell Evaluation Technology Research Association, Tokyo, Japan;; 4 DMPK, Research and Early Development Cardiovascular, Renal and Metabolism, BioPharmaceuticals R&D, AstraZeneca, Gothenburg, Sweden;; 5 Physicians Committee for Responsible Medicine, Washington DC, USA;; 6 Boehringer Ingelheim Pharma GmbH & Co. KG, Non-clinical Drug Safety, Biberach, Germany;; 7 Federal Agency for Medicines and Health Products, Brussels, Belgium;; 8 BfArM, Bonn, Germany;; 9 Javelin Biotech, Inc, Woburn, MA, USA;; 10 Clinical Pharmacology and Safety Sciences, R&D, AstraZeneca, Cambridge, UK;; 11 US Food and Drug Administration, Center for Food Safety and Applied Nutrition, College Park, MD, USA;; 12 InSphero, Schlieren, Switzerland;; 13 Novartis Institutes for BioMedical Research Chemical Biology & Therapeutics, Basel, Switzerland;; 14 Massachusetts Institute of Technology, Cambridge, MA, USA;; 15 Emulate Inc., Boston, USA;; 16 Center for Alternatives to Animal Testing, Bloomberg School of Public Health, Johns Hopkins University, Baltimore, MD, USA;; 17 Philip Morris International R&D, Neuchâtel, Switzerland;; 18 CN Bio Innovations Ltd., Welwyn Garden City, UK;; 19 Wyss Institute for Biology Inspired Engineering, Harvard University, Boston, USA;; 20 National Institute of Advanced Industrial Science and Technology (AIST), Tokyo, Japan;; 21 Japanese Center for Validation of Animal Methods, Tokyo, Japan;; 22 Beiersdorf, Hamburg, Germany;; 23 Center for Alternatives to Animal Testing-Europe, University of Konstanz, Konstanz, Germany;; 24 National Center for Safety Evaluation of Drugs, National Institutes for Food and Drug Control, Beijing, P.R. China;; 25 Fraunhofer Institute for Interfacial Engineering and Biotechnology IGB, Stuttgart, Germany;; 26 Faculty of Medicine, Eberhard Karls University Tübingen, Tübingen, Germany;; 27 National Center for Toxicological Research, FDA, Silver Spring, MD, USA;; 28 Nortis Inc., Seattle, WA, USA;; 29 Texas A&M University, College Station, TX, USA;; 30 Bayer, Investigational Toxicology, Berlin, Germany;; 31 National Center for Advancing Translational Sciences, National Institutes of Health, Bethesda, MD, USA;; 32 M.M. Shemyakin & Yu.A. Ovchinnikov Institute of Bioorganic Chemistry, Russian Academy of Sciences, Moscow, Russia;; 33 National Research University Higher School of Economics, Russia;; 34 Russian Ministry of Production and Trade, Moscow, Russia;; 35 Universiteit Leiden, Leiden, The Netherlands;; 36 Institute for Human Organ and Disease Model Technologies, Eindhoven, The Netherlands;; 37 MIMETAS BV, Leiden, The Netherlands;; 38 Daiichi Sankyo Co., Ltd., Tokyo, Japan;; 39 F. Hoffmann-La Roche Ltd, Roche Innovation Center Basel, Switzerland;; 40 AxoSim, Inc., New Orleans, LA, USA

## Abstract

The first microfluidic microphysiological systems (MPS) entered the academic scene more than 15 years ago and were considered an enabling technology to human *in vitro* (patho)biology and, therefore, to provide alternative approaches to laboratory animals in pharmaceutical drug development and academic research. Currently, the field generates more than a thousand scientific publications per year. Despite the MPS hype in academia and by platform providers, which say this technology is about to reshape the entire *in vitro* culture landscape in basic and applied research, MPS approaches neither have been widely adopted by the pharmaceutical industry yet nor have they reached regulated drug authorization processes.

Here, 46 leading international experts from all stakeholder groups - academia, MPS supplier industry, pharmaceutical and consumer products industries, and leading regulatory agencies - analyzed challenges and hurdles along the MPS-based assay life cycle in the second workshop of its kind in June 2019. The main findings were that the level of qualification of MPS-based assays for a given context of use and communication gaps between stakeholders are the major challenges slowing industrial adoption by end users, which in turn is causing a regulatory acceptance dilemma. This report elaborates on these findings and proposes solutions by providing recommendations and a roadmap towards regulatory acceptance of MPS-based models, which will benefit patients and further reduce laboratory animal use in drug development. Finally, the potential of MPS-based human disease models to feed back into laboratory animal replacement in basic life science research is discussed.

## 1 Introduction

### 1.1 Definitions and terminology

*Microphysiological systems* (MPS) are microfluidic devices capable of emulating human (or any other animal species’) biology *in vitro* at the smallest biologically acceptable scale, defined by purpose. The application of fluid flow (dynamic) for the physiological nutrition of the tissues and the creation of microenvironmental biomolecular gradients and relevant mechanical cues (e.g., shear stress) is a major aspect of these systems, differentiating them from conventional (static) cell and tissue cultures. This review uses the term MPS exclusively for microfluidic systems. It is acknowledged that the term MPS in scientific literature is sometimes applied to *in vitro* systems lacking flow. Naturally, this holds especially true for systems mimicking the very early embryonal stage of human biology or other human tissues lacking blood perfusion *in vivo*, such as cartilage.

MPS is an umbrella term for a number of words used in the field to describe subsets of MPS-based models, which are the basis for the development of MPS-based methods, tests and assays. MPS-based models comprise organ models and disease models. The term *MPS-based organ model* or *organ-on-chip* stands for a fit-for-purpose microfluidic device containing living engineered organ substructures (functional unit(s)) in a controlled microenvironment, which recapitulate one or more aspects of the organ’s dynamics, functionality and (patho)physiological responses *in vivo* under real-time monitoring. *Organoid-on-chip, spheroid-on-chip* and *tissue chip* are subsets of the term organ-on-chip specifying that the organ model is an organoid, a spheroid or a tissue, respectively. The term *MPS-based multi-organ model* or *multi-or-gan-chip* refers to the combination of two or more different organ models within an MPS-based model emulating systemic organ interactions. The term *MPS-based disease model* is used for any single or multi-organ model mimicking representative elements of the pathophysiology of a disease of a given species, for example, humans. The terms *body-on-chip* and *human-on-chip* are used in scientific literature in the context of MPS-based models envisioned to emulate entire holistic physiological organismal homeostasis. The latter still are at the level of scientific hypothesis-based ideas, not yet translated into any functional prototype or solution. The same applies to the term *patient-on-chip*, which is used in this report for MPS-based models envisioned to emulate personalized, patient-specific organismal pathophysiology. MPS-based methods, tests and assays are used by different stakeholders at three levels of quality:

- The terms *method* or *test* are used in this report for those that are primarily used in academia for basic and applied research to make new discoveries in a trial and error fashion. They are supposed to be reproducible scientific methods and tests according to common research standards. Knowledge and scientific publications are the prime outcome from this level of quality of MPS technologies.
- The term *qualified assay* is used in this report for those fit-for-purpose assays that have been adopted by and integrated into end user industries for candidate development and assessment and, therefore, have been optimized regarding their degree of standardization. Mechanistic understanding of the mode of action and adverse outcome pathways of new leads and investigative data for failed candidates are two examples of the outcome from this level of quality. The data support internal preclinical portfolio decision-making within the end user industries and can become part of an investigational new drug (IND) file or investigational medicinal product dossier (IMPD).
- The term *validated assay* is used in this report for those assays in a specific context of use that have been validated by end users in a setting relevant to regulatory approval processes for new medicines or consumer products. The outcome of this level of quality are assays finally introduced into International Council for Harmonisation of Technical Requirements for Pharmaceuticals for Human Use (ICH) or Organisation for Economic Co-operation and Development (OECD) guidelines.

For clarity regarding the terms *qualification* and *validation*: According to the US Food and Drug Administration (FDA)’s toxicology roadmap^1^, “*high-quality data, a thorough, unbiased, and transparent scientific review process, and confidence in the tools used to demonstrate safety and assess risk*” is critical to FDA’s ability to reach sound regulatory decisions and retain the public’s trust. “*FDA must be able to evaluate the applicability, limitations, relevance, reliability, reproducibility and sensitivity of a test or series of tests (performance standards) to confirm that they have been appropriately validated or qualified. Current formal approaches to validation involve lengthy and expensive processes that may not be necessary for all uses of a particular test. Rather than validation, an approach that the FDA frequently takes for biological (and toxicological) models and assays is qualification. Within the stated context of use, qualification is a conclusion that the results of an assessment using the model or assay can be relied on to have a specific interpretation and application in product development and regulatory decision-making. The term context of use refers to a clearly articulated description delineating the manner and purpose of use for the tool (when and how it will be used). Adequately specifying the context of use is often a difficult first step towards qualification and regulatory acceptance of new methodologies. Qualification also identifies the boundaries of the available data that adequately justify the use of the tool. Models and assays should be suited for a purpose and, in that context, they will have different applicability, assumptions and limitations. Once a new model or assay is considered qualified by the FDA for a specific context of use, industry and other stakeholders can use it for the qualified purpose during product development, and FDA reviewers can be confident in applying it without needing to review the underlying supporting data again*.”^1^

For the sake of simplicity, we have used the terms academia, MPS suppliers, end users and regulators for the four interested MPS stakeholder groups. In this report, the term academia stands for any nonindustrial institution performing MPS-based basic or applied research. The term *MPS supplier* comprises commercial providers and vendors of MPS-based devices, biological models, methods, tests and assays. The term *end user* describes those industries that adopt MPS equipment and MPS-based assays to support regulatory authorization of new medicines or consumer products, such as the pharmaceutical, biotech and consumer industries and contract research organizations (CROs) active in that field. The complexity of a model and the need for adaptation of an assay may influence whether a platform is to be transferred to the pharmaceutical industry or whether a fee-for-service model of a CRO is envisaged at end user level. The term *regulator* stands for all agencies and regulatory bodies responsible for the authorization of new medicines or consumer products in the respective geography of the world, such as FDA, European Medicines Agency (EMA), China Food and Drug Administration, Russian Ministry of Production and Trade and others. The term *developer* is used in this report for any person involved in discovering, inventing or improving MPS devices and MPS-based models, methods, tests and assays. Developers are represented in all four stakeholder groups, including regulators where regulatory science activities contribute to the improvement of MPS technologies. The term *regulatory science* is used for the science of developing new tools, standards, and approaches to assess the safety, efficacy, quality and performance of regulated products.

### 1.2 How to make preclinical drug testing predictive for human exposure?

The preclinical selection of drug candidates using laboratory animals and conventional *in vitro* cultures is not fail-safe, as compounds fail in clinical trials due to efficacy and safety concerns. However, the limited value of animals is illustrated by the enormous safety fail record of Phase I clinical trials. It should be noted that humans do not predict humans well either, or there would be few drug failures due to clinical safety in Phase II trials. A compilation of combined data on the attrition of drug candidates from AstraZeneca, Eli Lilly and Company, GlaxoSmithKline and Pfizer illustrated this dilemma: It revealed an attrition rate of 25% of the investigated drug candidates that was related to clinical safety in both Phase I and Phase II trials (Waring et al., 2015).

**Fig. 1:**
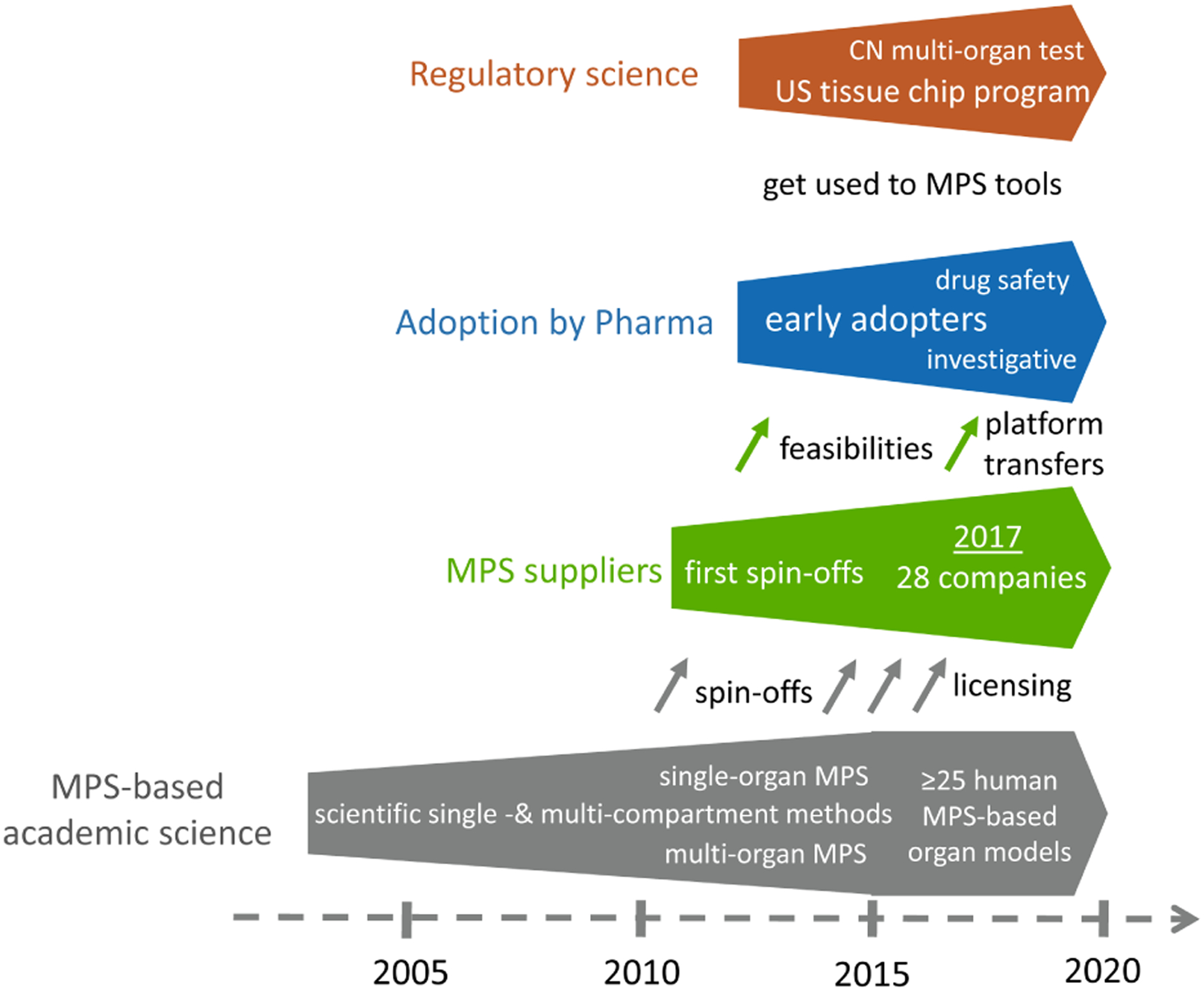
Historical sketch of the establishment of the MPS stakeholder community Grey and green arrows - impact of academia and MPS suppliers on other stakeholders in the process of development, transfer and use of MPS-based models and assays.

It is felt that utilizing a human-based, complex system has the potential to improve predictivity. MPS-based models bear the potential to emulate human biology at the smallest biologically acceptable scale as defined by purpose. The application of fluid flow for physiological nutrition of the organ models creates physiological biomolecular gradients and relevant mechanical cues (e.g., shear stress), mimicking the human situation. Therefore, validated MPS-based context of use assays might become a predictive alternative to existing preclinical tests or at least reduce the use of animals. A vibrant MPS stakeholder community consisting of the four stakeholder groups has been developed stepwise over the last 15 years (Fig. 1).

MPS developments started more than 15 years ago in academia with a wide range of inventions and tools based on single and multi-organ models and methods, the highlights of which are detailed in Section 2.1. In consequence, a vibrant MPS supplier industry developed from scientific labs. Prime examples are TissUse from the Technische Universität Berlin, Emulate from the Wyss Institute for Biologically Inspired Engineering in Boston, Mimetas from Leiden University, and Nortis from the University of Washington. Other suppliers licensed MPS technologies from academia. Prime examples are CN Bio licensing the PhysioMimix platform from the Massachusetts Institute of Technology (MIT) in Cambridge, MA, InSphero licensing the multi-tissue plate platform from the ETH in Zurich, and Hesperos using the technologies developed at Cornell University and at the University of Central Florida. A survey in 2017 identified that there were already 28 MPS suppliers serving different segments of the market (Zhang and Radisic, 2017). An ever-rising number of companies has entered the field since. The MPS supplier industry started with an array of business models ranging from supplying devices and chips to research labs, followed by feasibility studies for MPS-based models and methods for end user industries and, finally, transferring qualified MPS-based assay platforms to the pharmaceutical industry for routine in-house use. Early adopters began to apply MPS-based methods and assays for investigative purposes and drug safety testing, respectively, as described in more detail in Section 2.2. Finally, the FDA has been intensively involved in the US tissue chip program since 2011 in the framework of a regulatory science initiative, and Chinese regulators have been gaining scientific experience with MPS-based methods since 2014.

**Fig. 2:**
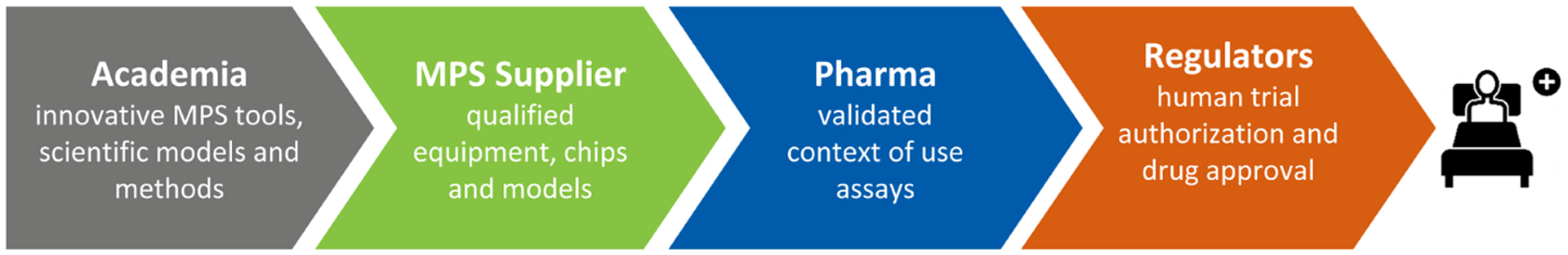
Life cycle of an MPS-based assay Academia-driven MPS inventions are translated into qualified MPS equipment and chips by the supplier industry. Developers of all four stakeholders create MPS-based models, methods and tests. The pharmaceutical industry subsequently selects a model for a specific purpose and validates the respective MPS-based context of use assay to test safety and efficacy of novel drug candidates or advanced therapies. These data support clinical trial authorization and, consequently, final approval for use in patients.

However, despite their disruptive potential and a more than 15-year history, the current life cycle of MPS-based assays, illustrated in Figure 2, is still in its infancy. The life cycle consists of four elements: i) academic invention and model development, ii) tool creation and model qualification by supplier industries, iii) qualification of a fit-for-purpose assay and its adoption for candidate testing by pharmaceutical industries, and iv) regulatory acceptance of the predictive results of validated assays for a drug candidate for a specific context of use. Experts have identified qualification and validation to be the major challenges slowing industrial adoption and stakeholder communication gaps to be causing the regulatory acceptance dilemma. Section 3 elaborates on existing scientific challenges, industrial hurdles and the communication gap in detail, whilst Sections 4, 5 and 6 provide experts’ opinions on how to overcome these roadblocks.

Furthermore, workshop participants identify and describe the areas where MPS-based models, methods, tests and assays can make a significant difference in the near future in Section 7. Finally, in Section 8, experts make detailed recommendations for short- and mid-term actions in the field and sketch a 15-year roadmap into the future towards preclinical candidate drug testing and advanced therapy evaluation.

## 2 MPS research highlights in academia and MPS-based assay adoption by industry

MPS-based models, methods and tests already have made incredible progress from proof-of-concept studies to actual implementation in many research fields and commercial activities globally.

### 2.1 Research highlights - past and present

A few labs pioneered the development of tissue models on chips in the first decade of the 21^st^ century (Baker, 2011). The following decade produced prime examples of outstanding research initiatives and projects that have shaped the MPS landscape. Here, we summarize research and development highlights that resulted from prime projects and initiatives in the US and Europe.

**Fig. 3:**
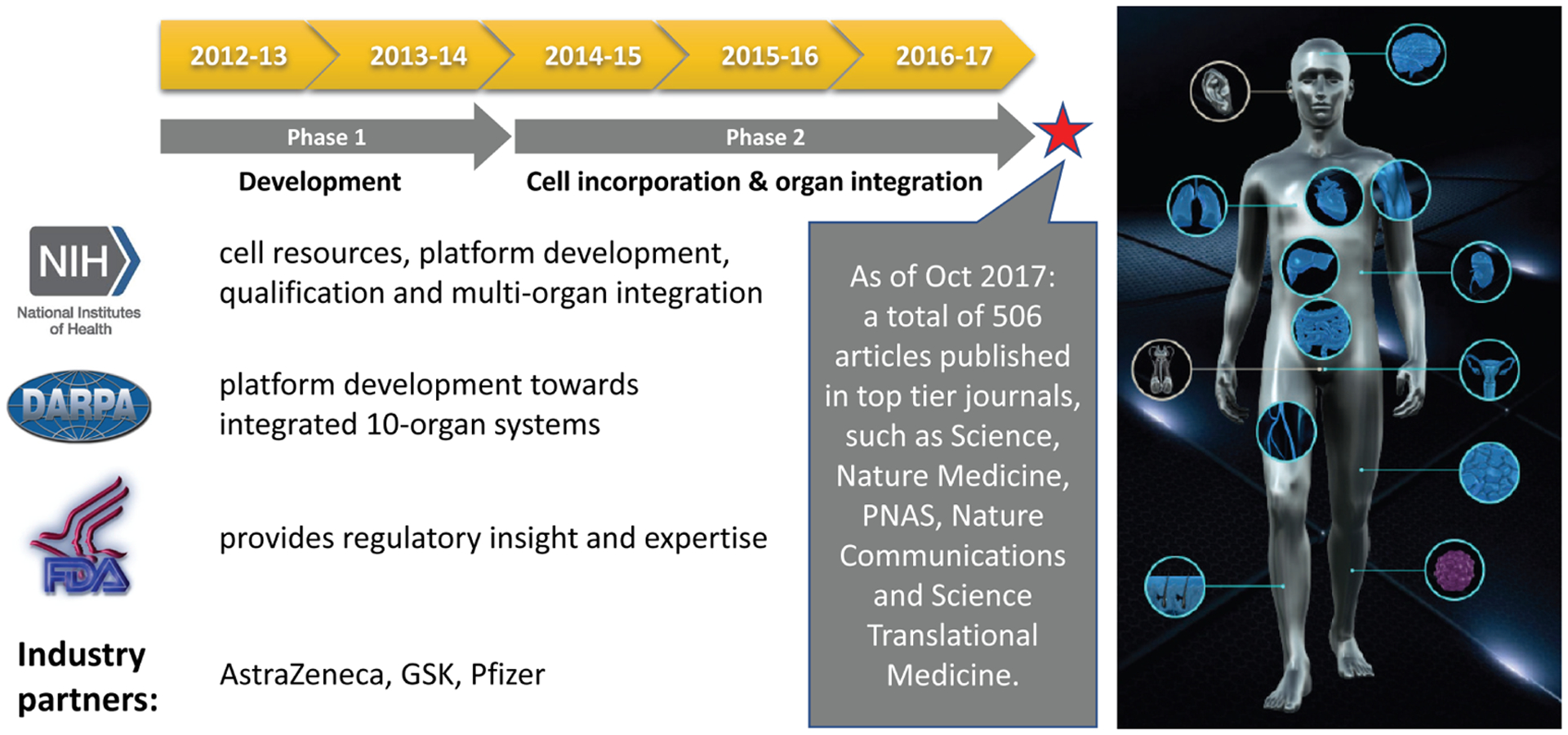
The US tissue chip program at a glance This FDA-DARPA-NIH MPS-based program aimed at developing *in vitro* platforms that use human tissues to evaluate the efficacy, safety and toxicity of promising therapies (adopted from Smirnova et al., 2018).

Inspired by the “lung-on-a-chip”, the first organ chip with tissue-tissue interfaces published in *Science* by the Wyss Institute (Huh et al., 2010), US National Institutes of Health (NIH) and FDA co-funded the Advancing Regulatory Sciences initiative (Low and Tagle, 2017a) to spur translational work in the regulatory sciences. One of the awardees was the team at the Wyss Institute, who aimed to develop a “heart and lung micromachine”. In 2012, the US Defense Advanced Research Projects Agency (DARPA) created a program “*to support the development of a systemic MPS platform, capable of mimicking the structure and function of at least ten major human organ systems using human cells and tissues, and which were to remain collectively viable in microfluidic culture conditions for at least a month, sufficient for safety and toxicity testing of candidate drugs*”. Donald Ingber’s team at the Wyss Institute and Linda Griffith’s team at MIT were the beneficiaries of that program. Simultaneously, the NIH, led by the National Center for Advancing Translational Sciences (NCATS), joined efforts with FDA and DARPA to support the development of MPS that mimic the structure and function of an array of individual major human organ systems using human cells and tissues. This program aimed for the same performance criteria for each of the MPS-based organ models (Fig. 3) and resulted in more than ten individual human organs and tissue chips being developed and described in more than 500 publications by the time it ended in 2017 (Low and Tagle, 2017b; Tagle, 2019).

In order to build confidence in MPS as a tool for drug development, NCATS partnered with FDA and IQ Consortium MPS Affiliate (see Box 1) to gain regulatory and industry input for its utility and to develop a validation set of compounds, biomarkers and assays that are salient for drug development. Members of the IQ Consortium recently published recommendations for *in vitro* model development and assay qualification of lung and skin models to facilitate their wider adoption for use within the pharmaceutical industry (Ainslie et al., 2019; Hardwick et al., 2019).

Towards this end, NCATS awarded two Tissue Chip Testing Centers (TCTCs), the Translational Center of Tissue Chip Technologies for Quantitative Characterization of Microphysiological System Technologies at MIT and TEX-VAL, the Texas A&M Tissue Chip Validation Consortium at Texas A&M University as well as a central database center for chip-based data, the Microphysiology Systems Database Center at University of Pittsburgh Drug Discovery Institute, that would take MPS platforms and cell sources from tissue chip developers and independently replicate published findings of the various tissue chips, assess their robustness, portability of the technology, develop best practices, and provide input for further improvement of the devices^2^ (Livingston et al., 2016; Low and Tagle, 2017b).

Failure to demonstrate efficacy is the most frequent cause of early termination of clinical trials, accounting for more than 60% of drug attrition (Hwang et al., 2016; Fogel, 2018). By incorporating advances in stem cell biology, genome editing, microfabrication and microfluidics, tissue chips can capture the pathophysiology of many human diseases and conditions (Low and Tagle, 2016). NIH, through its Tissue Chips for Disease Modeling and Efficacy Studies program,^3^ is currently supporting studies to develop *in vitro* disease models using primary tissue or induced pluripotent stem cell (iPSC)-derived patient cell sources on tissue-/organ-on-chip platforms, validate the disease relevance of these models, and test the effectiveness of candidate drugs on these models. A current focus of NIH in promoting MPS for disease modeling includes micropathophysiological systems of rare disorders and complex diseases such as type II diabetes, Alzheimer’s disease and dementia-on-chip. NIH also is supporting research into underdeveloped and extremely complex tissue systems, such as immune-system-on-chip, and nociception-, addiction- and over-dose-on-chip. NCATS is spearheading an initiative on the use of tissue chips for “clinical trials”-on-chip that will inform clinical trial design and implementation in precision medicine.

NCATS’ partnership with the International Space Station U.S. National Laboratory, formerly known as the Center for the Advancement of Science in Space, has a two-fold goal: 1) To understand the role of the environment, particularly microgravity, on human health and diseases as it relates to accelerated aging and to translate those findings to improve human health on Earth, and 2) to further innovate tissue chip technology through miniaturization and automation of the instrumentations that support the chips. For the former, it is known that symptoms of accelerated aging such as sarcopenia (muscle deterioration), osteoporosis, reduced cardiopulmonary function and immune senescence occur after pro-longed exposure to microgravity, however, these physiological changes are reversible when astronauts return to Earth.

The US Environmental Protection Agency (EPA) recently published a strategic plan as a response to the Frank R. Lauten-berg Chemical Safety for the 21^st^ Century Act, which updates the Toxic Substances Control Act (EPA, 2018). The main focus of the EPA’s activities following this strategic plan is the promotion and establishment of new approach methodologies for regulatory risk assessment, of which MPS should be a part. The EPA subsequently announced the elimination of all mammal study requests and funding by 2035 (EPA, 2019).

There are several examples of MPS used successfully in hard-to-study populations (rare diseases, pediatrics, pregnancy) and/or with an outcome which was missed in the animal model. One example among many MPS-based research projects across the US is the ongoing research at Wyss Institute, developer of multiple organ-on-chip models, beginning with the well-known lung alveolus chip and extending to include models of lung small airway, small intestine, large intestine, kidney glomerulus, kidney proximal tubule, liver, bone marrow and blood-brain barrier among others. A few recent, clinically relevant examples include the recapitulation of clinical responses to cigarette smoke measured at the cellular, molecular and transcriptomics levels in a human small airway chip (Benam, 2016a); demonstration of drug and radiation toxicities using clinically relevant drug and radiation doses; PK profiles for a drug currently in human clinical trials using a human bone marrow chip (Chou et al., 2018); and replication of species-specific (rat, dog and human) hepatotoxicities using liver chips created with cells from all three species (Jang et al., 2019). The Wyss Institute team also created human intestine chips lined with cells from patient-derived organoids (Kasendra et al., 2018); cultured complex human gut microbiome within it for multiple days by creating transepithelial hypoxia-gradient-on-chip (Jalili-Firoozinezhad et al., 2019) and modeled mitochondrial cardiomyopathy associated with Barth syndrome, a rare genetic condition, using a heart-on-chip with cardiomyocytes derived from patient- and genetically-engineered iPSC (Wang et al., 2014). Atchison et al. (2017) recently developed a blood vessel MPS to study the Hutchinson-Gilford progeria syndrome, a rare, accelerated aging disorder, recapitulating the key features of the disease and modeling drug responses. Glieberman et al. (2019) established synchronized stimulation and continuous insulin sensing in a microfluidic human islet-on-chip model designed for scalable manufacturing.

MPS research highlights in Europe have resulted from a number of national initiatives in the past few years. The German GO-Bio program on multi-organ bioreactors^4^ initiated by the Technische Universität Berlin generated a number of prime examples for the use of single and multi-organ chips. Co-culture of human models of healthy liver and skin (Wagner et al., 2013), liver and neuronal tissue (Materne et al., 2015), liver and pancreatic islets (Bauer et al., 2017), intestine, vasculature and liver (Maschmeyer et al., 2015a), and intestine, liver, skin and kidney (Maschmeyer et al., 2015b) were established to evaluate physiological cross-talk of the organ models and test primary and secondary toxicity of compounds. A co-culture of a human skin model with a tumor was developed for the simultaneous evaluation of safety and toxicity of anti-EGFR antibodies (Hübner et al., 2018). Finally, the program resulted in a PBPK-compliant four-organ chip hosting autologous intestine, liver, neuronal and kidney models differentiated from iPSC of a single individual donor for ADME (absorption, distribution, metabolism and excretion) profiling and toxicity testing (Ramme et al., 2019).

The Dutch Institute for Human Organ and Disease Model Technologies (hDMT) (see Box 2) and the Netherlands Organ-on-Chip Initiative,^5^ among others, published the following scientific research highlights: Scalable MPS to model three-dimensional blood vessels (de Graaf et al., 2019); inflammatory response and barrier function of iPSC-derived endothelial cells in a microfluidic chip (Halaidych et al., 2018a,b); Cytostretch, a silicon-based modular customizable organ-on-chip platform (Gaio et al., 2016); thrombosis-on-chip model (Westein et al., 2013; Jain et al., 2016; Costa et al., 2017) and prediction of toxic side-effects (Barrile et al., 2018); high-throughput model for perfused 3D angiogenic sprouting (van Duinen et al., 2019); cancer-on-chip model for the tumor microenvironment in metastasis (Sleeboom et al., 2018).

### 2.2 Examples of MPS application by the pharmaceutical industry

Over the past few years, the pharmaceutical industry has been increasingly assessing various MPS-based models, methods and assays from the supplier industry. Contract testing or the internal use of MPS-based assays are the drivers for those assessments. Some of the models have been established in the pharmaceutical industry and are used for internal decision-making at various stages in the drug development cycle. An anonymized survey among the workshop participants from end user and MPS supplier industries and among the IQ Consortium showed that areas of successful application include the entire value chain in drug development, ranging from discovery to preclinical and clinical development (Tab. 1).

Examples of assays that are currently used for internal decision-making include a liver-pancreas disease model, a gut epithelium and a blood vessel model for target identification and validation studies during the early discovery phase. Regarding preclinical development, a bone marrow-chip, a blood-brain-barrier-chip, an intestinal model for uptake studies and a lung-on-chip were mentioned. One example where an MPS-based assay is currently used during clinical development is a gut chip to clarify a potential mode of action-related intestinal toxicity. Galapagos discloses the use of the OrganoPlate (Trietsch et al., 2017; van Duinen et al., 2019) system for modeling scleroderma and inflammatory bowel disease (Beaurivage et al., 2019) both to understand disease biology and for compound evaluation. Novo Nordisk discloses that it is using vasculature models in the MIMETAS OrganoPlate for early target validation and identification. An undisclosed pharmaceutical company uses perfused kidney proximal tubules (Vormann et al., 2018) and blood vessels (van Duinen et al., 2017) in the OrganoPlate system to study pharmacokinetics and pharmacology of proprietary compounds. Outside of the drug development realm, Philip Morris International uses a 3D human microvessel-on-chip system that models key cardiovascular disease-related inflammatory mechanisms involved in the initiation of atherosclerosis in the context of the preclinical program for systems toxicological risk assessment of consumer products (Poussin et al., 2020).

However, most work is done on exploratory studies and model establishment outside of regular pharma portfolio work. Therefore, detailed information on the use and performance of MPS models in the pharmaceutical industry often cannot be shared as it is part of ongoing drug development programs. Thus, the sharing of experiences in a precompetitive manner, including approaches on how to characterize and qualify assays, would certainly be highly desirable, help advance the whole field, and result in mutual benefit for all users and developers in the community.

## 3 Scientific challenges, industrial hurdles and communication gaps for MPS

### 3.1 Challenges and hurdles faced by developers and suppliers

MPS developers are still facing a variety of scientific challenges in emulating human biology at a level sufficient to truly predict all aspects of the mode of action, safety and efficacy of new drug candidates or advanced therapies. Bioengineering was the foundation of MPS and paved the way for the exploration of a steadily growing number of different approaches on how to recapitulate complex biology in a dish. However, a number of challenges remain.

While basic aspects of various organs have been modeled and combined to form multi-organ chips, the most challenging parts of organ physiology, such as a closed vascularization and innervation of existing organ-on-chip models, are still missing. The lack of the vascular system is of special significance as it impedes the addition of a systemic immune system. Immune cells circulating between the organ equivalents and on-chip immune organs are vulnerable to nonuniformity in shear stress and prone to accumulate in small openings and gaps within the devices. However, innate and adoptive on-chip immune responses are of importance, for example, to study inflammation on-chip or effects of biopharmaceuticals. Metastatic tumor invasion studies, similarly, require the monitoring of cell trafficking in and out of a closed vascular system. The modeling of immunocompetent tumor microenvironments on-chip thus will advance when a closed vasculature is achieved.

Another challenge that is occasionally forgotten is the solid, constant source of good-quality cells. While there are many commercial resources for cell lines, a handful of iPSC-derived models and some primary cell types, different primary cells originating from the same organ or donor-matched cells in good quality and with a continuous supply often are not guaranteed. It is needless to state that a highly versatile technical setup only makes sense if the cells used in it also meet that degree of complexity and quality. Therefore, the cell source needs to be an integral part of the business proposition in order for a developer to make an investment into validating the system. This requires lengthy and often cumbersome licensing negotiations, limiting fast progress. The mushrooming of IP in the stem cell biology space adds to the challenge. In addition, multiple cell types are typically needed for a proper MPS approach, putting crucial consideration on royalty stacking provisions in order to maintain a viable commercial proposition. Therefore, even if the benefit of MPS for future implementation is evident, long-lasting cash from convinced industry players, brave long-term investors, and governmental or other funds are required for the development, qualification and commercialization of MPS.

The MPS supplier industry is facing challenges in the commercial arena. Aspects to consider include the fact that the business case of each supplier can be very different depending on where in the drug development process their solution potentially applies. The different stages along the value chain come with their particular needs regarding flexibility, physiological relevance, robustness and throughput. Furthermore, the willingness or need and the time available for users to explore and invest into additional, potentially very costly approaches with unclear benefits also varies greatly at different steps of drug development. Questions are typically highly focused on a specific endpoint in therapeutic disease areas. Regarding target identification and validation, more physiologically relevant systems could provide significant added value, while an MPS-setting may not apply for screening and selecting potent hits from a library. Very targeted assays that are well established are typically used during drug development stages where early characterization tests for ADME and toxicity come into play and, depending on the modality, larger numbers of candidates undergo testing and optimization. At advanced stages, where a handful of candidates are characterized for selection of a potential clinical candidate, MPS systems could support addressing potential human-relevant organ toxicities that are difficult to mimic in simple cell-based screens. These examples underpin the need for developers of such systems to weigh the investment required for validation of MPS against defined market size, limiting the type of developments that result in a viable proposition. In addition, an early engagement with drug development teams to assess where there are fields of application is strongly recommended to avoid establishing solutions where there is no problem.

### 3.2 Hurdles for adoption of MPS systems in pharmaceutical industry

Drug development is a lengthy, cumbersome and especially complex regulated procedure where costs and pressure to deliver in a particularly competitive environment are extremely high. Therefore, a pharmaceutical drug development team will not put the progress of a promising compound at risk by generating data that might be harmful in nonmandated systems. Only models that are critically needed in order to progress the compound and in which researchers have confidence that they will produce relevant and informative data will be used. Doubling data with, for example, existing and new models is feasible to validate a new approach, but the potential future benefit of the new approach has to be evident in order to justify such costly extra efforts.

One can, thus, conclude that there are limited incentives for the pharmaceutical industry to implement new, perhaps still experimental models that do not add obvious value in a classical drug discovery cycle, particularly when the application is far down the pipeline. Therefore, incentives for using MPS on compounds during drug discovery are highest a) when the MPS system can aid in rescuing a molecule that is at risk, b) for testing a back-up molecule if the frontrunner has failed for a specific issue the model can recapitulate, and c) if existing validated models are considered irrelevant for the drug and, therefore, the bar to apply new tools is lower.

Another incentive lies within early drug discovery projects where MPS could become an important asset for exploring new targets and treatment paradigms. At this stage, models reflecting relevant disease states would be of interest, especially if the target is unknown or not well defined. Models that are fed by, for example, patient-derived tissue could have great potential.

Recent years have seen an explosion of MPS concepts in the literature and a slowly but steadily growing number of companies as system providers. Although promises are typically high, convincing solid datasets underpinning these claims often do not exist or lack the breadth and depth required to trigger the interest of drug development teams. On the other hand, pharmaceutical companies would need to make significant investments in both time and non-portfolio budget to evaluate all the different emerging approaches to find out which could add value. Therefore, pharmaceutical companies have become hesitant regarding larger investments and involvement in collaborations or consortia. Consequently, only a small number of MPS approaches have undergone thorough characterization and pressure-testing in a real drug development environment.

The validation of MPS systems is typically performed as a combined effort of system providers and end users against existing models, including suboptimal cell culture models and animal experiments. Particularly for the latter, the validation would require MPS versions of the respective animals from whom legacy data is available. Toxicologists especially want to complete the parallelogram rat *in vitro* - rat *in vivo* - human *in vitro* - human *in vivo*. To date, public funding has been focused on the human *in vitro* component, leaving it up to the pharmaceutical industry to fund the rat version. A coordinated approach of a head-to-head evaluation of MPS-based liver models of human, rat and dog origin was recently accomplished as a result of a supplier-pharmaceutical industry collaboration (Jang et al., 2019). In an ideal situation, the MPS models would be exclusively compared to human data, however, clinical data for the detailed physiological parameters of interest is often not available.

Concluding, the major hurdle for industry to adopt MPS often lies in the technical immaturity of many of the systems, which results in complicated handling, minimal throughput, poor reproducibility, and often a lack of robustness. This is particularly the case for the latter aspects between established commercial suppliers and academic start-ups. These issues pose a true challenge for investing in MPS for application in an industry setting and, therefore, the field is encouraged to balance claims on the performance of a new system that could create unrealistic expectations. Seeking customer feedback at the start and throughout the development process for a new MPS model is highly advisable, also to avoid investment into solutions where there is no problem or a solution that does not solve the problem. A growing number of professional CROs who are specialized to use qualified MPS-based models and assays for contract testing of pharmaceutical compounds would accelerate adoption of MPS-systems by end users.

### 3.3 The stakeholder communication gap

**Fig. 4:**
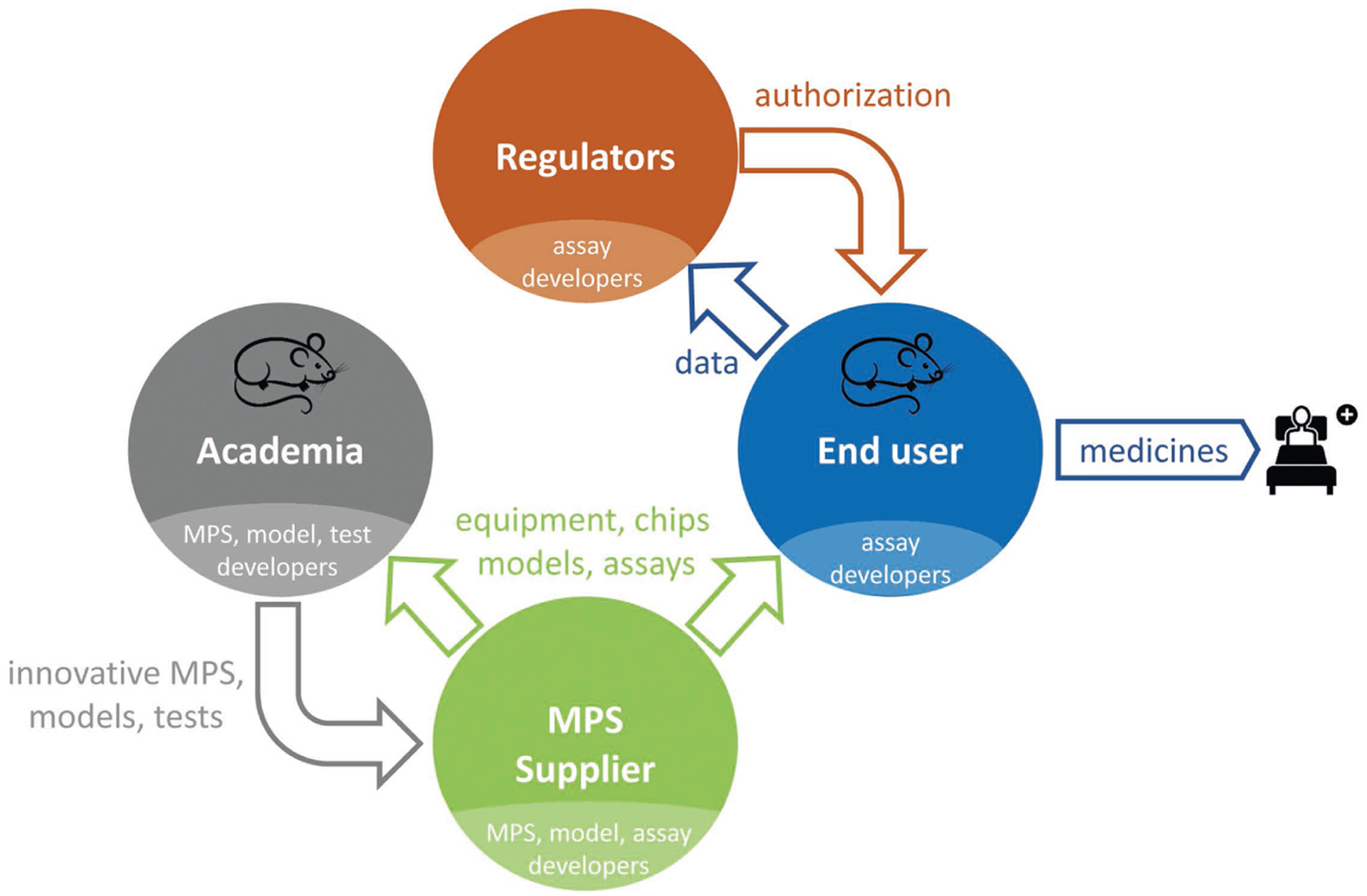
Established stakeholder interaction channels MPS devices, chips, models and methods are provided to end users and academia for data generation by the supplier industry. End users (pharmaceutical industry and CROs) are translating the methods into qualified assays for internal decision-making and use the data for clinical trial submissions, eventually resulting in authorization by regulators. Academia develops new MPS solutions that are absorbed by MPS suppliers. All four stakeholders consist of developers of MPS technologies.

During the course of the workshop, stakeholder experts analyzed the role and impact of each stakeholder group on the MPS-based assay development life cycle and the current interaction channels between the stakeholders (Fig. 4). They identified an urgent need to improve stakeholder communication in order to drastically enhance the quality and adoption of MPS-based assays.

An early engagement of end users to clarify their needs is required, as those needs are often unclear to developers. Similarly, a lack of agreed measures of success among different customers complicates model establishment and qualification. Guidance on clear criteria, for example, regarding a given organ system and the physiological parameters to be measured, would be welcome. The absence of agreement and harmonization sometimes becomes evident even within one company, where one unit within an end user company might be unaware of similar work already being undertaken by another unit within the company. It is also necessary to bring conservative and more innovative groups within one entity to an agreement.

Success stories showing a clear impact on the portfolio are critical in order to increase the adoption of MPS systems in routine drug development. A problem for the MPS developer community is that such portfolio success stories are typically not shared as the information around ongoing programs is confidential. Another aspect to be considered is that the individual contribution of an MPS-based system to decision-making during drug development might be difficult to define as decisions are rather reached based on a larger collection of endpoints stemming from different types of experiments.

Due to the high visibility of MPS, there is a significant risk of overselling or overpromising. It is important to distinguish between early proof-of-concept studies and true application in routine use to keep the interest and excitement of end users high. The intensification of information exchange between the different stakeholders early on would generally streamline research activities towards models needed in the pharmaceutical industry, facilitate model qualification, and prevent false expectations.

## 4 Global networking strategies - solving the communication gap

Improving communication regarding MPS may change the mind-set and help end users to embrace this new technology. Sharing success stories publicly, for example, will aid in the adoption of MPS and stimulate consolidation of the field. By incentivizing end users to make MPS case studies publicly available, the scientific community may be guided in refining their systems. Similarly, developers are encouraged to engage early on with MPS suppliers and end users to define their needs and specify the added value a system might bring to them. The respective area of application of the devices should then match the corresponding fit-for-purpose and context of use. In the following subsections, workshop participants highlighted prime existing initiatives, programs and networks that provide platforms for communication between stakeholders at a national level. However, global networking and exchange of stakeholders is still in its infancy and requires coordinated actions.

### 4.1 The US tissue chip program - a prototype for inclusive stakeholder networking

The NIH and FDA’s Advancing Regulatory Sciences initiative joined NIH’s efforts with FDA and IQ Consortium described in Section 2 to establish a solid US communication platform between academia, the end user industry and regulators at a national level. The US MPS supplier industry has been included through the TCTCs, which invited suppliers to apply with products and assays for evaluation.

In order to gain experience and knowledge with MPS technology in anticipation of seeing this technology in regulatory applications, the FDA has brought several different MPS technologies into its laboratories. FDA signed a Cooperative Research and Development Agreement with Emulate Inc., a commercial MPS supplier and the Wyss spin-off company, to use their organ-on-chip technology as a toxicology testing platform. It aims to beta test and conduct research using their liver system and the “Human Emulation System” (Emulate, Inc., 2017; Fitzpatrick, 2017). The Center for Drug Evaluation and Research in the Division of Applied Regulatory Science has the liver-on-chip from CN Bio, another commercial MPS and MIT spin-off company, in its lab. It is also working with Dr Kevin Healy on a heart-lung MPS. FDA’s Biologics Lab is working with CuriosisT to develop organoid models. FDA’s National Center for Toxicological Research has partnered with TissUse to develop an MPS containing organoids for two tissues linked by a microfluidic circuit for drug toxicity testing. FDA’s Medical Counter Measures (MCM) program is working with the Wyss Institute to develop models of radiation damage in lung, gut, and bone marrow organs-on-chips for candidate MCM testing. The work is part of the FDA Predictive Toxicology Roadmap announced in 2017.^1^

The Center for Alternatives to Animal Testing (CAAT) at Johns Hopkins University proposed a public private partnership for performance standards for MPS (P4M), where MPS performance standards will be discussed with stakeholders to accelerate regulatory acceptance (Smirnova et al., 2018). CAAT entertains secretariats for an MPS and Systems Toxicology program and Good Cell Culture Practice program, which serve as brokers between different end users by promoting MPS in the form of workshops and supporting guidance documents, such as the OECD Guidance Document (GD) on Good *In Vitro* Method Practices (OECD, 2018), a recommendation on reporting standards (Hartung et al., 2019) and a Good Cell Culture Practice (GCCP) document for iPSC and MPS (Pamies et al., 2017, 2018). A guidance document for GCCP 2.0 is in preparation.

### 4.2 Recent European initiatives for stakeholder networking

A number of national networks have been created in Europe in addition to the Dutch hDMT described in Section 2.1. The UK Organ-on-a-Chip (OoC) Technologies network^6^ is a Technology Touching Life initiative, jointly funded by the Medical Research Council, the Engineering and Physical Sciences Research Council and Biotechnology and Biological Sciences Research Council, designed to capture, inspire and grow UK research activity in the organ-on-chip research field. The network is open to industrial, clinical and academic partners and aims to i) develop a vibrant multidisciplinary research community, bringing focus to the varied organs-on-chips and *in vitro* model research activity in the UK, ii) facilitate interdisciplinary and inter-sectoral research collaborations to develop the next generation of organ-on-chip research solutions, and iii) train, support and inspire the next generation of outstanding leaders in organ-on-chip research. Furthermore, a Finnish Centre of Excellence in Body-on-Chip Research^7^ and a Norwegian Hybrid Technology Hub and Convergence Environment organ-on-chip and nano-devices activity^8^ have been established.

More recently, multiple integrative European MPS focused activities have started to establish a communication and collaboration framework for advancement of the field in Europe.

#### ORCHID

The 2-year Horizon 2020 Future and Emerging Technologies Open project *Organ-on-Chip In Development* (ORCHID^9^) started in 2017 with the goal of creating a roadmap for organ-on-chip technology and of building a network of academic, research, industrial and regulatory institutions to move organs-on-chips from laboratories into general use to benefit the citizens of Europe and beyond. The ORCHID Consortium is a collaboration between seven partner organizations from six European countries: from the Netherlands the Leiden University Medical Center (coordinator), the Institute for Human Organ and Disease Model Technologies (hDMT, see Box 2), and the Delft University of Technology (TU Delft); from France the Commissariat à l’Energie Atomique et aux Energies Alternatives; from Belgium the imec; from Germany the Fraunhofer Institute for Interfacial Engineering and Biotechnology (Fraunhofer IGB); and from Spain the University of Zaragoza. It engages an international advisory board of world-renowned experts.

Two workshops were held with experts from academia, cosmetics and the pharmaceutical industry, representatives of patient organizations, ethics schools, biotechnology companies, innovation hubs and regulatory agencies. The results of bibliographical, bibliometric and market analyses and of expert interviews, combined with the insights and conclusions from the workshops, resulted in two publications. The first publication describes current unmet needs, key challenges, barriers and perspectives of this technology and recommendations for defining a European organ-on-chip roadmap (Mastrangeli et al., 2019a). The other publication reports the six specific building blocks for the roadmap that have been defined, including priorities, methods and targets for each block and the facilitating role of the European Organ-on-Chip Society (EUROoCS)^10^ (Mastrangeli et al., 2019b), being another outcome of the ORCHID project. The economic impact of organs-on-chip (Franzen et al., 2019), new business models and training needs have also been identified.

During the final ORCHID meeting, the European roadmap was presented to a broad audience of end users, regulators, clinicians, developers, policymakers and patient representatives. There is consensus on the major impact that EUROoCS will have in the deployment as well as the actualization of each of the building blocks. Since qualification and standardization will accelerate organ-on-chip technology implementation, activities in this direction will have the highest priority. Among the first are the design and implementation of a European organ-on-chip infrastructure with testing, training and data centers, resulting in independently qualified and characterized models and the development of open technology platforms to enable customized solutions for specific applications. This will guide end users in selecting the technology best suited to their purpose and provide the training needed to create success. EUROoCS will initiate and catalyze these challenging processes.

#### MSCA-ITN EUROoC

The interdisciplinary training network for advancing organ-on-chip technology in Europe (MSCA-ITN EUROoC^11^) started in 2018. EUROoC created a trans-European network that consists of application-oriented researchers well trained in both the development and the application of organ-on-chip technologies. Due to the fast development of the field, a multidisciplinary background is required for the next generations of researchers entering this field. Basics in biology and microfluidic chip engineering are the cornerstones. EUROoC offers the first holistic European training program in the field. It gathers participants from chemistry, biology, medicine, engineering and physics in a network. It consists of companies (three small and one medium size enterprises), ten academic entities and two regulatory bodies. It is EUROoC’s mission to educate the next generations of scientists from different fields on all aspects of organ-on-chip development. In addition, a major focus in education will be utilization of organs-on-chips, including commercialization and aspects of regulatory acceptance.

#### EUROoCS

Collaboration between all stakeholders is key to the further acceptance, development and implementation of organ-on-chip technology. A growing network of research groups in more than 17 countries has recently been formed in Europe. In addition to the Netherlands, many countries, including the United Kingdom, Scandinavia, Belgium and Israel, have started to link organ-on-chip players in their countries. This will create strong collaborations throughout Europe and beyond and, therefore, create the basis for a European Center of Excellence on human organs-on-chip.

The surge of European activities has led to the launch of the European Organ-on-Chip Society^10^ as an independent, not-for-profit organization established to encourage and develop research in the field. Furthermore, it provides opportunities for advancing and sharing knowledge. Individual researchers and other persons interested in organ-on-chip technology can become members of the society. Benefits include the annual conference, with plenty of opportunities for interaction between young researchers, and access to a digital platform on organs-on-chip. The platform supports exchange of expertise and research projects between members. It initiates discussions with others and enables new collaborations. EUROoCS will provide a platform for interaction between all parties who are involved in the implementation of the organ-on-chip roadmap strategy. With the support of EUROoCS, the organ-on-chip community will be built further in order to bridge the gap between end users, developers and regulators. EUROoCS organizes the annual EUROoCS conference on challenges in the process of designing, fabricating and implementing organs-on-chip. The EUROoCS conference gathers the research leaders in this emerging field with a special focus on training young and upcoming scientists.

As a result of these activities, the European Commission has picked up the technology and integrated it in multiple H2020 work programs, such as the Nanotechnologies, Advanced Materials, Biotechnology and Advanced Manufacturing and Processing program (cf. “H2020-DT-NMBP-23-2020: Next generation ‘Organ-on-Chip’”).

### 4.3 The Japanese AMED-MPS project

**Fig. 5:**
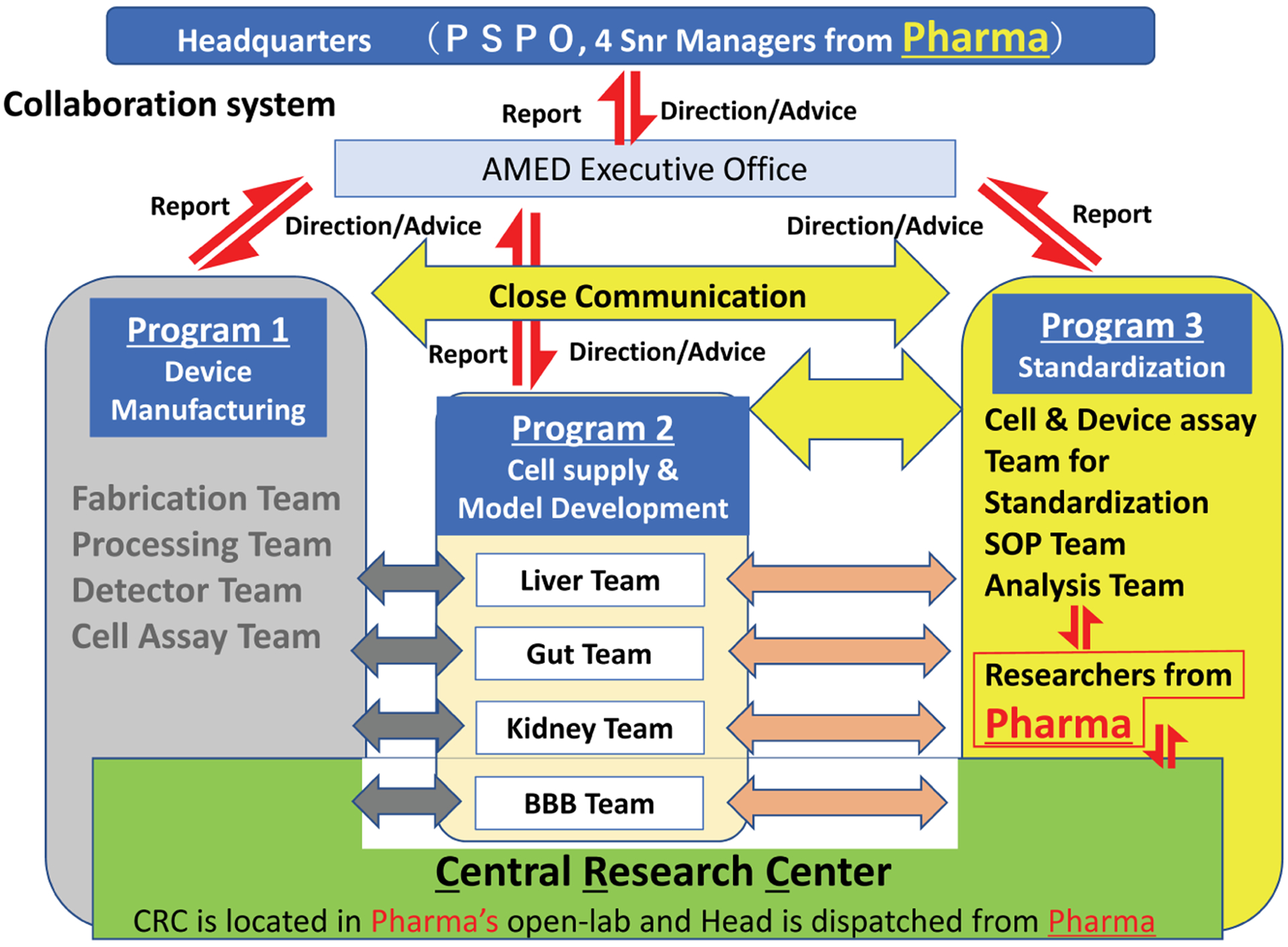
The Japanese AMED-MPS program at a glance The interdisciplinary research teams are developing four human organ models and the Central Research Center is uniting researchers and end users to accomplish the program.

In 2017, the national MPS project AMED-MPS was launched in Japan (Fig. 5). It is supported by the Japanese Agency for Medical Research and Development (AMED) and consists of three research programs, a central research center, and a headquarters for establishing close communication among academic developers and end users.

The main research program is the Organ Model Development Research Program, focusing on cell supply and MPS model development of four organs: liver, gut, kidney and blood-brain barrier. Industrial programs include the Device Manufacturing Research Program for developing manufacturing technology for industrial products and the Standardization Research Program for developing standardization of MPS models for quality control and regulatory development. It is noteworthy that senior managers and researchers in pharmacokinetics and safety/toxicity fields from domestic pharmaceutical companies participate in the project as members of the decision-making body and research partners.

In order to bridge the gap between developers and end users, the central research center, closely collaborating with manufacturing and standardization program members, conducts research and development to transfer newly developed MPS-based models to end users for implementation. Therefore, the program recapitulates the early communication arrangements of the US program; the active involvement of regulators and MPS suppliers are next challenges.

### 4.4 Communication and outreach

US stakeholders established a first productive communication platform between academia, end users and regulators, which served as a prototype for other geographies. However, the MPS supplier industry is still not fully involved. The workshop participants therefore concluded that there is no effective communication platform in place that includes all four stakeholder groups at a global level and developed a number of recommendations outlined in detail in Section 8 (Box 4). In brief, the establishment of a global international society on MPS with continental sections, such as that developed in Europe^10^, which can coordinate activities and collaboration on a smaller scale, is envisioned. The international society will maintain the overview of the main activities and new developments in the field worldwide and share and advance knowledge to help early integration of end users’ requirements into early development to maximize the outcome and use of a given MPS-based model, method or assay. The society will be responsible for biannual meetings focusing entirely on MPS and for organization of the specialty sessions at the international conferences, such as Society of Toxicology Annual Meeting and the World Congress on Alternatives and Animal Use in the Life Sciences. Patient groups should be involved with the goal of communication and outreach and to increase the involvement of end users.

## 5 Qualification of MPS - how to address the major challenge for industrial adoption?

### 5.1 The traditional *in vitro* assay validation process

Reproducibility assessment and qualification of MPS-based scientific models, methods and tests is (or should be) a standard procedure for academia, the MPS supplier industry and end users, resulting in a qualified assay. Validation of MPS-based assays in the pharmaceutical industry or formal validation as defined by OECD GD 34 (OECD, 2005), including, for example, ring trials, is typically restricted to the generation of data for regulatory authorization. It should provide regulators with adequate information on the suitability of an assay validated for a specific context of use. Such validated MPS-based assays should be distinguished from models, methods and tests described previously, as they include a way to derive the test result as defined in the test protocol and its data analysis procedure. OECD validation standards may differ from the FDA’s regulatory qualification standards. Therefore, validated MPS-based methods are segment-specific, e.g., for chemicals or pharmaceuticals.

The validation process of *in vitro* assays (Hartung et al., 2004; Leist et al., 2012) is intended to provide confidence in test results by determining reproducibility and relevance for a given purpose, thus defining where the test may or may not be applied, and by presenting an account of test characteristics, such as precision, limit of detection, accuracy, specificity, sensitivity, robustness and transferability. The need for validation of *in vitro* assays became evident when alternatives to animal use started gaining momentum in the 1980s. The classical definition of validation in this context was proposed in 1990 at a workshop of the European Centre for the Validation of Alternative Methods (ECVAM) and the European Research Group for Alternatives in Toxicity Testing (Balls et al., 1990): “*Validation is the process by which the reliability and relevance of a new method is established for a specific purpose*.” The modular approach to validation (Hartung et al., 2004) introduced further improvements, such as the use of existing data, leaner designs, applicability domains and performance standards. The modular approach, a consensus between ECVAM and its US counterpart, the Interagency Coordinating Committee on the Validation of Alternative Methods (ICCVAM), introduced the aspect of scientific validity and referred to the prediction model: Validation is a process in which the scientific basis and reproducibility of a test system, and the predictive capacity of an associated prediction model, undergo independent assessment.

The OECD GD 34 (OECD, 2005) harmonized validation processes, giving guidance on “Development, Validation and Regulatory Acceptance of New and Updated Internationally Acceptable Test Methods in Hazard Assessment.” It incorporated the Modular Approach among others. It defined validation as follows: Test method validation is a process based on scientifically sound principles by which the reliability and relevance of a particular test, approach, method or process are established for a specific purpose. Reliability is defined as the extent of reproducibility of results from a test within and among laboratories over time, when performed using the same standardized protocol. The relevance of a test method describes the relationship between the test and the effect in the target species and whether the test method is meaningful and useful for a defined purpose, with the limitations identified. In brief, it is the extent to which the test method correctly measures or predicts the (biological) effect of interest, as appropriate. Regulatory need, usefulness and limitations of the test method are aspects of its relevance. New and updated test methods need to be both reliable and relevant, i.e., validated.

It is important to note that the validation process is under constant evolution, as it is adapting to the different assessment needs and learning over time (Hartung, 2007). Hartung et al. (2013), for example, suggested a framework of mechanistic validation principles to suit the mechanistic tests of Tox21. These have not yet been broadly applied but lend themselves as broad principles in the validation of MPS.

### 5.2 Challenges of validating MPS-based assays

**Fig. 6:**
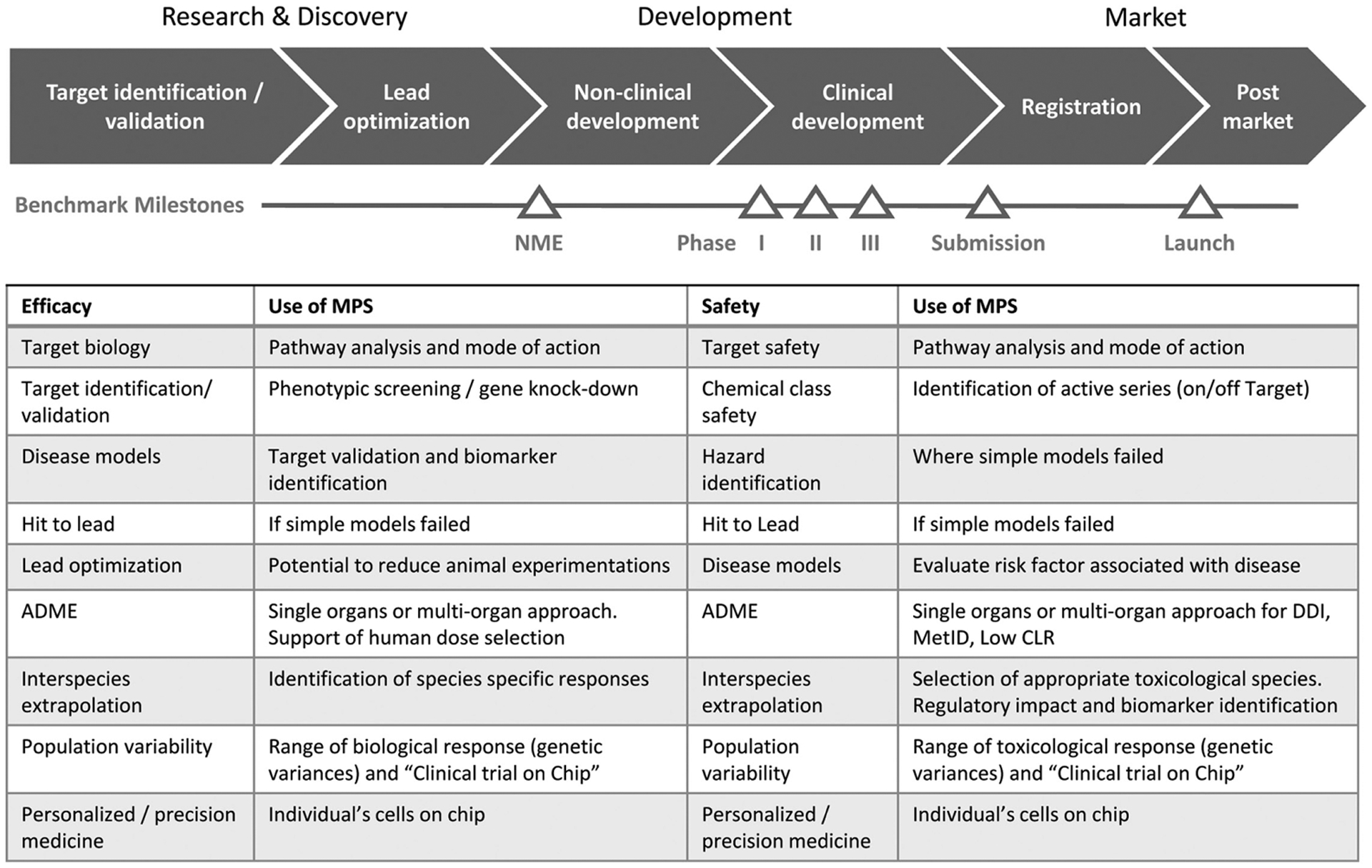
MPS-based assay application aligned to the drug discovery and development life cycle

MPS-based assays are complex *in vitro* approaches that are expected to be relevant for several purposes. Within drug discovery, this includes target validation, mechanistic analyses and risk assessment (see Fig. 6).

Acceptance of these systems does not rely solely on matching the relevant biology with a specific purpose and ensuring reproducibility of results but also on the quality control of the various components in MPS development (Tab. 2). This pertains to the assurance of quality materials, devices (specifications), biological materials, sensor/readout specifications, auxiliary equipment, standard operating procedures (SOPs), and documentation of results (see Tab. 2).

Starting with the cells, MPS-based models usually adopt cellular co-cultures and, therefore, individual quality criteria need to be established for each cell used. MPS-based models are increasingly being established with iPSC. Such cells are derived through complex differentiation processes involving a series of growth factors, each of which needs consideration in terms of quality assurance.

Further challenges are realized when considering that MPS-based models are enabling end users to derive predictive complex functional biological endpoints. As a result, MPS-based models are typically created with more than one compartment to allow cellular organization to be representative of the tissue. Additionally, each compartment has been designed to be true to the cellular microenvironment and, as such, control of temperature and pH is critical. The presence of multiple compartments also creates more complex *in vitro* biokinetics and often results in the adaptation of technologies such as microscopy (Peel et al., 2019).

Multi-organ systems present additional challenges, as the improved biological relevance afforded by the dynamic interaction of different organoids/tissues may come at the cost of increased complexity. Precise timing of cell culture and organoid formation, for instance, is critical to ensure equivalent maturity; the different organoids/tissues usually have different cell culture medium requirements, and any validation conducted using individual MPS-based models would need to be repeated for multi-organ systems to account for differences introduced by bringing the models together.

Engineering plays a critical role in MPS-based model development, and each of the components needs to be documented and controlled, as they can impact the performance and sensitivity of the model. This includes microfluidics and integrated sensors. Factors out of user control, such as changes to the supply chain and batch quality, may also influence outcome. The lack of platform standardization across MPS-based models results in multiple qualification steps and necessitates higher requirements for the training of personnel.

In many ways, MPS-based models emulate a higher complexity of human biology than 2D *in vitro* assays, therefore, traditional validation routes, such as ring trials, are less relevant. Ring trials are expensive, can take three to ten years, and the number of test compounds will be limited by throughput and high setup or operation costs for MPS-based assays. These systems also cannot be scaled up in the same way as 2D cell culture because there is a limit to the number of devices that can be assayed simultaneously. Furthermore, the associated IP for MPS-based models and assays exists typically in only a few laboratories and has to be managed accordingly by the MPS supplier industry to provide freedom to operate for end users. In general, having a stable supply chain for device construction (e.g., material supply, reliable cooperation with external suppliers, licenses) and assay setup (e.g., robust and long-term supply of cells and scaffolds, cell culture media and supplements) for MPS suppliers and end users is an essential prerequisite for device commercialization and assay validation.

### 5.3 International programs for testing/qualification of MPS-based assays

The wide adoption of MPS-based assays by end users has been hampered by a lack of information on the reliability and relevance of this technology when applied to “real-life” problems. Some efforts have been made to address the confidence gap through in-house or independent testing of the robustness and reproducibility of the MPS-based models, methods and tests (Livingston et al., 2016). Strategic roadmaps to bridge the gap between the innovators and end users through independent testing processes have been proposed by the IQ Consortium MPS Affiliate and NCATS (Livingston et al., 2016; Ewart et al., 2017) to build confidence in the utility of MPS-based assays. A report on “Using 21^st^ century science to improve risk-related evaluations” called for the promotion of fit-for-purpose validation and clearly defined the comparators and gold standards (National Academies of Sciences, Engineering, and Medicine et al., 2017). The committee noted that establishing the utility and domain of new assays, clearly defining how test results should be interpreted in terms of a positive/negative response, and developing performance standards for the assays under test that enable the evaluation of relevant adverse outcomes are key needs for MPS-based assays.

Indeed, the topic of the testing/qualification of complex *in vitro* models has been given much attention in the broader scientific and regulatory community since 2016. An EURL ECVAM survey of 646 individuals with awareness or good familiarity with complex *in vitro* models, including MPS-based models, methods and tests, representing diverse sectors in 36 countries was conducted in 2018 to get a better understanding of how best to establish the *in vitro* models’ validity for use in research and testing with a view to building end user confidence (EURL ECVAM, 2018). The survey showed that 65% of responders had already conducted some form of internal qualification of MPS, most using internal procedures, with only about 5% of these relying exclusively on some version of formal guidelines. The responders favored independent review of the complex *in vitro* systems by an almost 6:1 margin. Furthermore, 45% of the responders stated that establishing the validity of a complex *in vitro* model outside a context of use is possible and useful to increase its acceptance and use more broadly.

Several coordinated efforts have been undertaken recently to conduct the testing/qualification of MPS. Their outcomes form an important foundation for defining the general principles for the testing/qualification of MPS.

The TCTCs were established^2,12^ in the United States with funding from NCATS to provide a way to evaluate the performance of tissue chip platforms developed through the NCATS-funded Tissue Chip for Drug Screening program.^13^ Investigators from MIT and Texas A&M University are conducting the independent experiments with a diverse range of tissue chip platforms, and the University of Pittsburgh has developed a tissue chip database,^14^ where information from the testing of each organ platform is deposited. Examples of the outcomes from tissue chip testing by these centers are beginning to appear in the peer-reviewed literature (Sakolish et al., 2018). The work of these centers will advance the wider adoption of the technology by the pharmaceutical and biotechnology industries and regulatory agencies and assists with the transition of the technology into commercialization.

In Europe, the cosmetics industry is bound to the exclusive use of non-animal methods for the qualification of new ingredients by Regulation (EU) 1223/2009 (EC, 2009). The current *in vitro* assessment gap for compounds that become bioavailable after skin permeation, oral uptake or inhalation prompted Cosmetics Europe, the European trade association for cosmetics and personal care, to evaluate the benefits of MPS for human risk assessment regarding systemic toxicology. This is also in accordance with Cosmetics Europe’s Long Range Scientific Strategy (Desprez et al., 2018), which encompasses the evaluation of integrated systems by combining static and dynamic skin and liver 3D models (Wagner et al., 2013) within its toxicokinetic project. The integration of skin equivalents in MPS is expected to (partly) emulate skin barrier function and metabolism and to provide information on potential first-pass metabolism in the skin and its interaction with other tissues. The main goal of the project is to scrutinize the system’s suitability to provide risk assessment-relevant data. This includes exposure scenario-dependent effects on the metabolic fate of chemicals and the elucidation of potential alterations of the tissues’ metabolic capacity after longer-term repeated exposure. Proof-of-concept case studies were not limited to cosmetic ingredients, but rather featured compounds chosen to assess the system. A main aspect of the approach is to rate the quality and validity of resulting information by analyzing the intra- and inter-laboratory reproducibility after transfer of the method to another lab. In addition to its own activities, Cosmetics Europe is a partner in the EU-ToxRisk program in evaluating the use and benefit of four-organ chip technology for ADME and toxicodynamic analyses of case study compounds. The use of MPS for safety assessment is also being implemented in several cosmetic companies to evaluate the application of different models for different purposes.

In Japan, the Stem Cell Evaluation Technology Research Association (SCETRA)^15^ is a not-for-profit research and development organization that specializes in supporting the development and practical application of advanced technologies using human stem cells, most recently MPS-based models. SCETRA is a partnership of pharmaceutical companies, government agencies, device manufacturers and other stakeholders that aims to shorten the research and development cycle of novel technologies, including tissue chips, and improve their successful use by end users.

These efforts collectively not only bridge the gaps between MPS developers from all four stakeholders one model at a time, they are also working to define general principles for testing and qualification of MPS-based methods and tests. A standardized workflow for MPS-based model testing and qualification, for example, may be conducted in a tiered manner: Material transfer, testing of the flow and of drug-binding to the devices, replication of the experiments performed by the developers, and testing of new drugs selected in partnership with the end users. Another conceptual approach to the sequence of steps in testing/qualification of MPS-based methods and tests may include the testing of the device’s technical performance, physiological relevance to the organ/tissue it is meant to mimic, and its fit-for-purpose for drug or chemical testing.

The most common barriers to the successful transfer of MPS technology between laboratories in a reproducible manner are a model’s throughput, cost, accessibility of the endpoints that can be assessed using widely available equipment, and the availability of cells and other necessary reagents and materials to establish and maintain the tissue chip in a functional state.

In conclusion, the working group welcomed a number of wide-ranging international efforts aimed at promoting the use of MPS-based assays by conducting testing and qualification exercises. Regardless of the intricacy of the model, method or test and its level of biological complexity, the following are the defining basic principles of MPS-based assay testing/qualification: The model, method or test and related technical equipment (i) should be transferable; (ii) should replicate published results; and (iii) should be applied to the purpose of need in a precisely defined context of use.

### 5.4 Roles and responsibilities of stakeholders in the qualification process

**Fig. 7:**
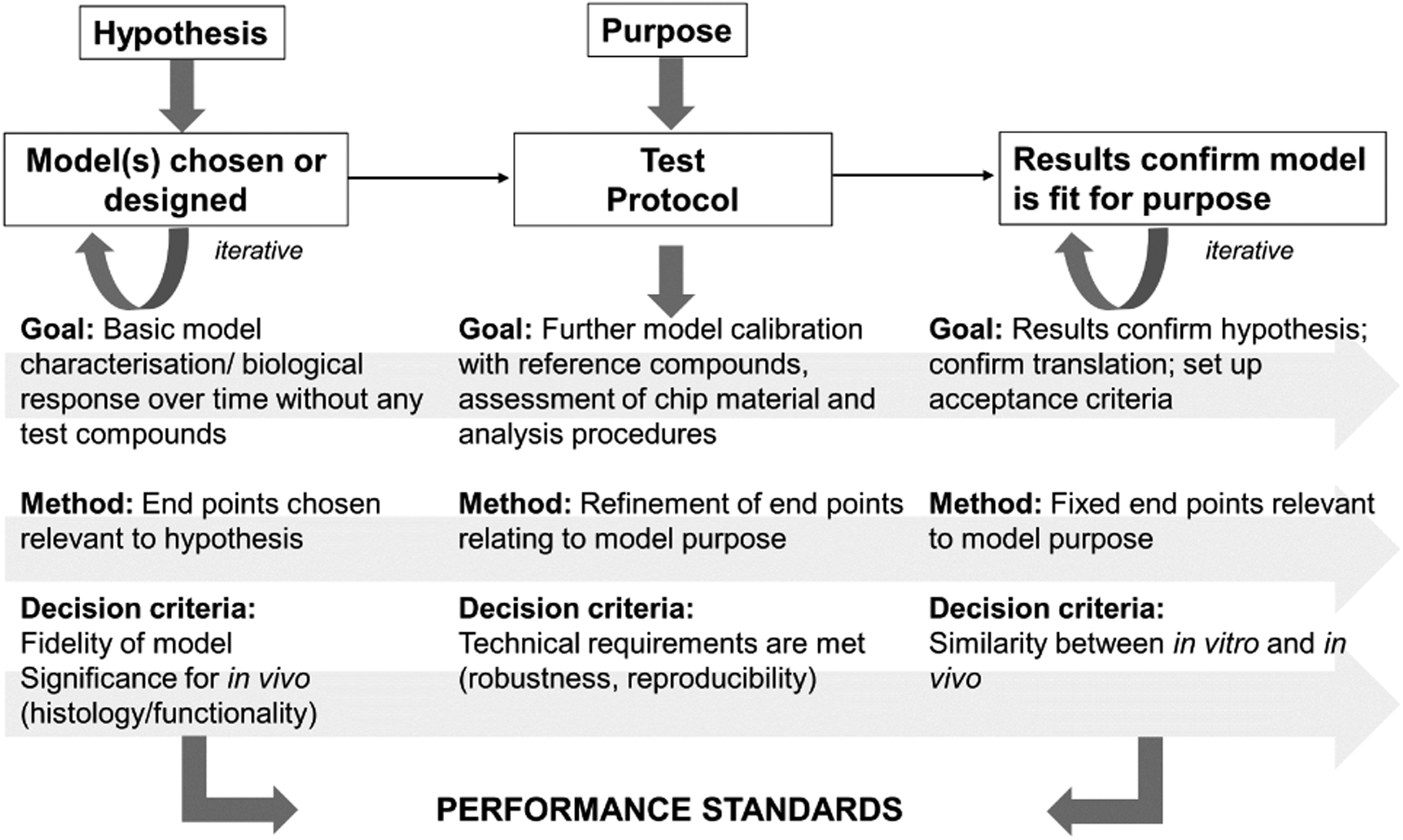
Steps towards MPS-based assay qualification which will define the performance standards

As described in Section 5.2, each component of an MPS-based assay should be considered during the qualification process. Figure 7 provides an outline of necessary steps.

Participants in the process of model/test development and qualification are required to think beyond the scope of their specific expertise and keep the whole process of model/test development in mind to ensure that their stakeholders will be able to meet their respective quality criteria and performance standards. A policy of transparent exchange of information among developers of all stakeholders, MPS suppliers, end users and regulators is expected to facilitate mutual learning and eventually guarantee high quality at every level, ensuring relevant and reproducible results. Such an approach can be facilitated by a data exchange platform.

Comparable technical skills and uniform interpretation of SOPs are prerequisites for intra- and inter-laboratory reproducibility. To that end, clear and intuitive documentation of SOPs is essential. However, due to high complexity, some systems may require additional training and harmonization of processes, materials used, and data generation and analysis. In many cases, this may be achieved by joint development of tests by developers and end users. This need for harmonization of cell sources, SOPs, model transfer, lab hardware used to generate, for example, 3D tissues from cells, documentation of data analysis, and interpretation and potential training pertains explicitly to the evaluation of models by the TCTCs (see Section 5.3). Their evaluation of the transferability and robustness of the models should rate these aspects and is, consequently, an important indicator of the maturity/quality of the models, providing important input for developers, end users and regulatory bodies on the applicability of the corresponding model and the validity of its results. Programs such as Innovative Training Networks will further help to disseminate expertise and improve the application of models for different sorts of tests.

## 6 How to solve the regulatory acceptance dilemma?

Despite qualification and validation, a frequent argument justifying the lack of industrial use of MPS-based methods and tests in safety assessment is that regulatory agencies need to formally communicate that MPS-based methods and tests are accepted or indicate what data are needed to obtain regulatory acceptance. However, this is only partly true, since regulators, in turn, have noted that applicants have so far submitted very little or no MPS-based assay data to FDA or EMA. Therefore, experience with and confidence in these data cannot be gained by regulators.

It is a dilemma similar to that of gene expression data some 15 years ago, when FDA and EMA encouraged the submission of exploratory data sets under a mechanism originally termed “safe harbor” (Hackett and Lesko, 2003; EMA, 2006). Such exploratory data would become part of a knowledge database of the agency and would be shared with the public. It is usually not used in decision-making.

In addition, mature MPS-based assays have existed for only a few years and have been used in the meantime by only a few early adopters, whereas most pharmaceutical companies can be considered rather as mid-term to late followers. The technology will be incorporated into the safety or efficacy testing portfolio only after internal evaluation and the evolution of sufficient internal confidence (e.g., robustness, predictivity) in MPS-based assay applications, each qualified assay being accepted only for its specified context of use. Given the duration of the evaluation phase and the subsequent drug development cycle times of the preclinical phase, it follows that MPS-based assay data acquired only recently by early adopters will take at least three to five years until it eventually might be submitted as part of an IND application or a clinical trial authorization (CTA) submission. However, regulatory agencies and industry leaders have a role to play in speeding up acceptance and adoption.

Scientists at FDA and EMA are taking the initiative to gain experience with MPS-based assays through participation in this workgroup, through involvement in ongoing collaborations, such as the FDA’s participation in the tissue chip collaborations with the NIH and the DARPA, the FDA’s partnership with commercial platform suppliers to evaluate MPS-based assays for drug safety, and the application of MPS-based methods in FDA labs. While early adopters and regulatory agencies are building experience with MPS-based assays now, late followers are waiting for signals from the regulatory arena regarding use and acceptance.

Adopters at all stages would benefit from work towards regulatory acceptance by regulators. There are multiple options for regulators to contribute to the advancement of MPS-based methods and tests into qualified assays for regulatory application. Firstly, regulators could begin working to establish performance criteria for MPS that outline what regulators need to see in order to accept MPS *in lieu* of traditional testing. Secondly, and more broadly, regulators can work to establish a clear pathway for the evaluation of new methods that includes communication, such as guidance how to translate a scientific method into a valid assay, that will be useful within a specific context of use. It is currently possible to qualify a platform on a case-by-case basis within an application; the downside is that the approach may then be drug-specific and kept secret. However, such specific use cases could assist the development of a general guidance in a specific context of use. This would mean a two-way process where new information from users feeds into the regulatory process and *vice versa*.

To spur innovation and enable platform use for broader areas across multiple countries, it would be useful to convene a group of international regulators in the drug, device, food and biologics space to share knowledge of emerging sciences and technologies and work together to move the new approaches forward.

### 6.1 Establishing an environment of voluntary MPS data sharing and submission

Since regulatory agencies are not allowed to publish MPS data submitted in the course of a drug application, joint workshops, collaborations, for example, between industry, academics and regulators, regulator position papers and other forms of communication including agency guidance are the only possibilities to advance the adoption of the technology. In addition, the submitter of the MPS data should be encouraged to publish the pertinent data and the assessment results. This would also be true for results helping to characterize not only the valuable outcome but also the limits of a certain MPS.

Representatives of FDA and EMA expressed their interest in MPS data that was used to decide against putting a candidate compound forward for development, which could be presented in a joint workshop format. During the workshop, participating representatives of regulatory agencies stressed that all MPS data submitted would be evaluated according to its usefulness for risk assessment and regulatory decision-making. FDA has started an internal program to test several MPS platforms in order to bridge the gap in terms of time and experience.

FDA also has created a horizon scanning effort to prepare for new sciences and technologies that may impact regulatory centers in five to ten years. The public is requested to enter their ideas in an open docket.^16^ FDA has also organized two public meetings that will discuss some of the work being done within the FDA on alternative approaches including MPS. The first is the second meeting on the FDA’s Predictive Toxicology Roadmap^1^; the second is the FDA’s Science Forum.^17^

The EMA organized a stakeholder workshop under the heading: “First EMA workshop on non-animal approaches in support of medicinal product development: challenges and opportunities for use of micro-physiological systems” in 2017 (EMA, 2017). This workshop was targeted at the nonclinical development of medicinal products and aimed at mapping the current state of science in this field together with developing a common understanding of the benefits and limits of these methods between developers, users and regulators. In addition, this workshop, through an open dialogue with the stakeholders concerned, aimed at identifying the next steps that would foster regulatory acceptance of MPS in the near future and on a longer time scale.

The Chinese government has been actively advocating the innovation of medical technology to promote the development of the pharmaceutical industry. Many academic research institutions in China have independently developed a variety of organ chips (e.g., 3D microvascular models), but the process and design of microfluidic technology lacks industry specifications. The MPS-based test analysis is also limited to laboratory research, has not been applied in the pharmaceutical industry and has not entered the national regulatory evaluation process.

The National Institutes for Food and Drug Control (NIFDC) is a subordinate agency of the National Medical Products Administration. A prime mission of the NIFDC is to organize the international collaboration and cooperation in the field of testing and analysis of drugs, medical devices and cosmetics. The NIFDC signed a memorandum of understanding on regulatory science in the field of human-on-chip technologies with the Technische Universität Berlin to get hands-on experience with MPS-based approaches. A five-year collaboration starting 2015 was conducted in the NIFDC. At present, the NIFDC team has conducted toxicology endpoint-driven experiments for a human two-organ arrangement on a chip. More tests will be carried out in the field of toxicity and efficacy evaluation using multi-organ chips.

Several activities are recommended to increase the confidence in and comfort level of the new technology and the obtained data. Firstly, a working definition of MPS (see the Section 1.1) is necessary to delineate the system(s) under consideration. Libraries of commercially available reference compounds used in MPS for a specific context of use then need to be established jointly between users and MPS providers with the oversight of regulatory agencies. The data acquired should ideally be curated by an honest data broker as a neutral third party. Such activities require consortia and respective funding. The development of specific MPS testing centers financed by industry consortia is one approach. Alternatively, consortia could be formed as a public-private partnership, such as under the umbrella of the Innovative Medicines Initiative.

Based on the reference compound libraries, performance standards should be developed, where data already accrued by the system providers (e.g., internal quality control standards) could be integrated. Such an inventory would ideally be supported or driven by a global regulatory partnership. Table 3 provides an overview of the envisaged endpoints and data contained in such a library.

Many of these principles are not unique to MPS-based assays. Since the use of MPS-based assays is expected to entail a reduction of animal studies, essentially all relevant regulatory guidance documents on the acceptance of 3Rs testing approaches also apply to MPS-based assays (e.g., EMA, 2016).

### 6.2 Development of use cases in a regulatory context

Specific guidelines on MPS-based assay acceptance or inclusion of MPS-based assays in integrated approaches to testing and assessment (IATA) can be envisaged after acquiring sufficient regulatory experience a few years from now. Table 4 lists some of the challenges currently faced in the regulatory realm. These challenges can be overcome with a learning-by-doing approach involving regulatory science activities.

In the meantime, position papers, ideally aligned internationally among regulatory agencies, will be helpful for sponsors to learn how the agencies deal with the data based on case examples. Such case examples could also be of a theoretical nature and span the gamut of regulatory safety testing. During the workshop, for example, the participants discussed the potential use of MPS for de-risking in a specific case where a liver finding was observed in rats but not in dogs. Classically, after analyzing the available data, such as differences in target/off-target expression, differences in bioavailability and exposure, and species-specific metabolites, the sponsor could resort to testing the candidate in a second non-rodent species (e.g., the nonhuman primate) to assess the relevance of the rat data (or the lack thereof) for humans. As an alternative, the sponsor could apply a humanized MPS-based assay, which has been shown to depict the adverse effect observed in rats (e.g., cholestasis or necrosis) with reference compounds. Whether the platform can reproduce all potential liver-related endpoints regardless of what was seen in the rat is controversial. Another theoretical scenario could be the decision of the sponsor after obtaining the rat liver findings to base the selection of the nonrodent species exclusively on data using MPS from multiple species. Such an approach would be particularly valuable if the mechanisms of a species-specific event are well-described (e.g., peroxisome proliferation in rats) and can be shown in the respective species-specific MPS. The use of MPS for such purposes would be a direct contribution to the reduction of animal studies.

A focus on specific reduction or replacement scenarios could eventually lead to the inclusion of MPS into ICH guidelines. The latter could, for example, be envisaged in ICH M3(R2), Guidance on nonclinical safety studies for the conduct of human clinical trials and marketing authorization for pharmaceuticals (EMA, 2013). This guideline requires (in chapter 17) nonclinical combination studies to support clinical trials under certain circumstances. An MPS-based assay has shown significant advantages over animal studies (Sieber at al., 2018), particularly for the assessment of bone marrow toxicity after treatment with different combinations of oncological medicinal products. This supports the chance to replace such combination studies, especially in a case when significant knowledge about the mode of action of the individual compounds is available and different modes of action can be depicted in an MPS-based assay. On the other hand, a complete replacement of regulatory animal safety studies will only be possible if MPS-based assays can reproduce effects on roughly 50 to 60 organs and tissues not only individually but also in their interaction, a requirement which is hardly imaginable in the near future but remains a long-term goal. Finding situations for which MPS-based assays can provide added value over and above the current *in vivo* models will provide opportunities to contribute to regulatory safety and efficacy evaluation and help gain the confidence of the community.

### 6.3 Disease models in a regulatory context

The lack of concordance of animal disease models with human treatment outcome has been reviewed intensively (van der Worp et al., 2010). On the other hand, animal disease models are far less regulated by guidelines, even though the data are used in a regulatory context for estimating efficacious doses for clinical trials. The development of humanized MPS-based disease models has great potential, particularly in areas where the lack of concordance is well documented or where the target is not or insufficiently expressed in animal species. The lack of meaningful animal disease models for advanced therapies in immune-oncology and for some chronic diseases, such as in the field of neurodegenerative disorders, might speed up the adoption of humanized MPS-based assays and accompanying technologies in the pharmaceutical industry.

## 7 Areas where MPS can win in drug development and beyond

Clearly, the strongest incentive for the adoption of MPS systems in the pharmaceutical industry is given when a model offers recapitulation of biological processes that cannot be assessed using traditional cell culture models and where it is known that similar data from animal experiments would not be translatable and/or are irrelevant. The opportunity of MPS to provide physiological culture conditions to complex models makes them especially strong, such as the ability to understand new targets and treatment paradigms of multi-organ diseases and systemic effects. In the following, a number of niches are discussed where MPS systems could be particularly successful today and in the future.

### 7.1 Disease modeling

The establishment of human-relevant disease models is a highly unmet need. Preclinical models to study basic aspects of disease are still almost exclusively mouse models, complicating the translation to the clinic. While drug development has made major advances in assessing metabolism and pharmacokinetics and reducing toxicity early on to avoid subsequent surprises in clinical phases, a steadily increasing proportion of drug development programs fail because of lack of efficacy in patients, which underlines the poor human relevance of many of the disease models currently used. These models often include genetically modified mice that are used for the identification of promising drug targets and to perform pharmacological proof of concept studies. The ability to recapitulate aspects of disease in MPS by using human cells, i.e., from patient-derived tissue, has the potential to overcome some of the limitations described - provided prior thorough validation supported by real world patient data is used. In order to use MPS as human models in phenotypic screening for target discovery, their compliance with high throughput testing requirements needs to be established. The poor success rate in drug discovery could be overcome by employing complex, physiologically relevant assays early in development.

Areas where a physiologically relevant human cell model could be key is when the immune system comes into play, as this area is well-known to be regulated dramatically differently between species (e.g., rodents and humans). The complexity of many of the diseases and the numerous unknowns leading to a diseased phenotype in humans make it very challenging to establish a simplified reductionist cell model that can be efficiently exploited for disease modeling. Solid, confirmatory studies with representative compounds are required to test different hypotheses on a mode of action.

An additional aspect to consider is the use of such MPS-based disease models for early biomarker discovery. Having a truly translational biomarker in place early on that informs on target engagement and disease modification potential would be very beneficial for drug development.

### 7.2 Drug programs with minimal animal testing and “*in vitro* only” approaches

The use of conventional animal testing may be less relevant for some drug development areas if, for example, the species in focus are not considered to be responders. In some cases, no animal species may be relevant if very human-specific targets and respective drug molecules are used. There is a likelihood, particularly for large molecules that nowadays are highly engineered constructs, that not even the cynomolgus monkey, the animal species of choice for large molecule drug programs, is cross-reactive. For those cases, MPS systems recapitulating identified human target organs or representing key organs for efficacy and safety may be used to test and characterize drug candidates. By doing so, the approach into clinical testing based on the so-called “minimal anticipated biological effect level” may benefit significantly, for example, by determining safe starting doses using state-of-the-art MPS models.

MPS also could play a key role in rare disease indications. These programs naturally struggle with limited preclinical data and a small number of patients. Here, patient organizations with access to both data and tissue may collaborate with drug developers in academia or industry to establish relevant MPS-based disease models for drug testing.

### 7.3 Informing clinical trial design

Individuals differ substantially in their response to pharmacological treatment. MPS could be an important asset to embrace these differences. Based on previous clinical data from a given drug program or information from publications or preclinical investigations, potential side effects in a subset of patients can be anticipated. Similarly, depending on the pharmacological effect and disease-specific target expression variability, there are probably subpopulations that can benefit more or less from a given treatment. More specifically, both of the factors described suggest different dosing to maximize patient benefit. MPS-based models using patient-derived tissue may be used to test such hypotheses and define markers that can inform to stratify patient subpopulations. These then can be used to assess, for example, which patient subpopulations may be at risk and should receive, for example, a lower dose, less frequent dosing or should not receive a particular drug. On the other hand, the optimal dose for some patients may be higher than for others depending on, for example, target expression. Provided knowledge around these patient factors is available and/or hypotheses can be tested by using patient tissue, such approaches hold promise for improved early trial design. Along the same principles, MPS systems may be used to assess differences in drug response determined by ethnic diversity, gender or age.

### 7.4 Implementing successful models in other parts of the drug pipeline

Development and adoption of MPS models could be boosted tre-mendously in the applications mentioned above. This could help pave the way for broadening the spectrum of use of MPS to areas where there are currently fewer activities. Physiology-based MPS models providing at least a functional absorption barrier (intestine or lung equivalents), a metabolically competent human liver model and a functional excreting kidney equivalent, for example, might generate compound-specific ADME profiles. MPS-based target organ models might support compound-specific pharmacokinetic studies. Consequently, MPS-based assays involving such models might support preclinical pharmacokinetic and pharmacodynamic studies. It is anticipated that a panel of known target organ models will be developed that capture the most important hallmarks of compounds in preclinical evaluation.

### 7.5 Reducing and replacing animal studies

It has been frequently proposed that MPS bear the potential to replace regulatory *in vivo* studies that are mandatory for drug approval by the regulatory authorities. To achieve GLP (good laboratory practice) status, which is mandatory for regulatory studies and assures that the results are clearly reproducible, MPS require further maturation. Therefore, MPS might not replace a regulatory *in vivo* study in the short-term, as those tests are fundamental to a drug safety assessment, ensuring safe dosing in first-in-human trials. It would pose a significant challenge to drug development teams to negotiate with authorities not to perform any of these tests.

A more likely goal for MPS to meet is the replacement of pre-paratory and exploratory studies that are performed in animals before and during the regulatory GLP phase. Here, reliable *in vitro* models could play a key role in characterizing drug candidates and avoiding surprises in later phases. MPS could help overall with more informed decisions and can actually replace animal testing one-on-one for some of the pilot animal tests performed. The prerequisite for replacing these animal tests during the clinical candidate selection phase is the availability of good organ models for major organ toxicity with relevance to the human situation. As an example, two-organ combinations of liver and a secondary organ might be of interest when a small molecule drug is in the focus and if the metabolism of the compound is considered to play a key role.

The general preclinical development path currently involves testing in two animal species, a rodent and a nonrodent. In the UK, the NC3Rs have been looking at the inclusion of the second species in toxicity testing and what alternatives there may be. If regulatory agencies can make a decision solely on MPS data from species-related chips, this could spur the pharmaceutical industry to consider reducing laboratory animal usage in this field (Jang et al., 2019).

## 8 Recommendations, a roadmap towards and a long-term vision for accepted MPS-based models and assays

### 8.1 Recommendations

**Fig. 8:**
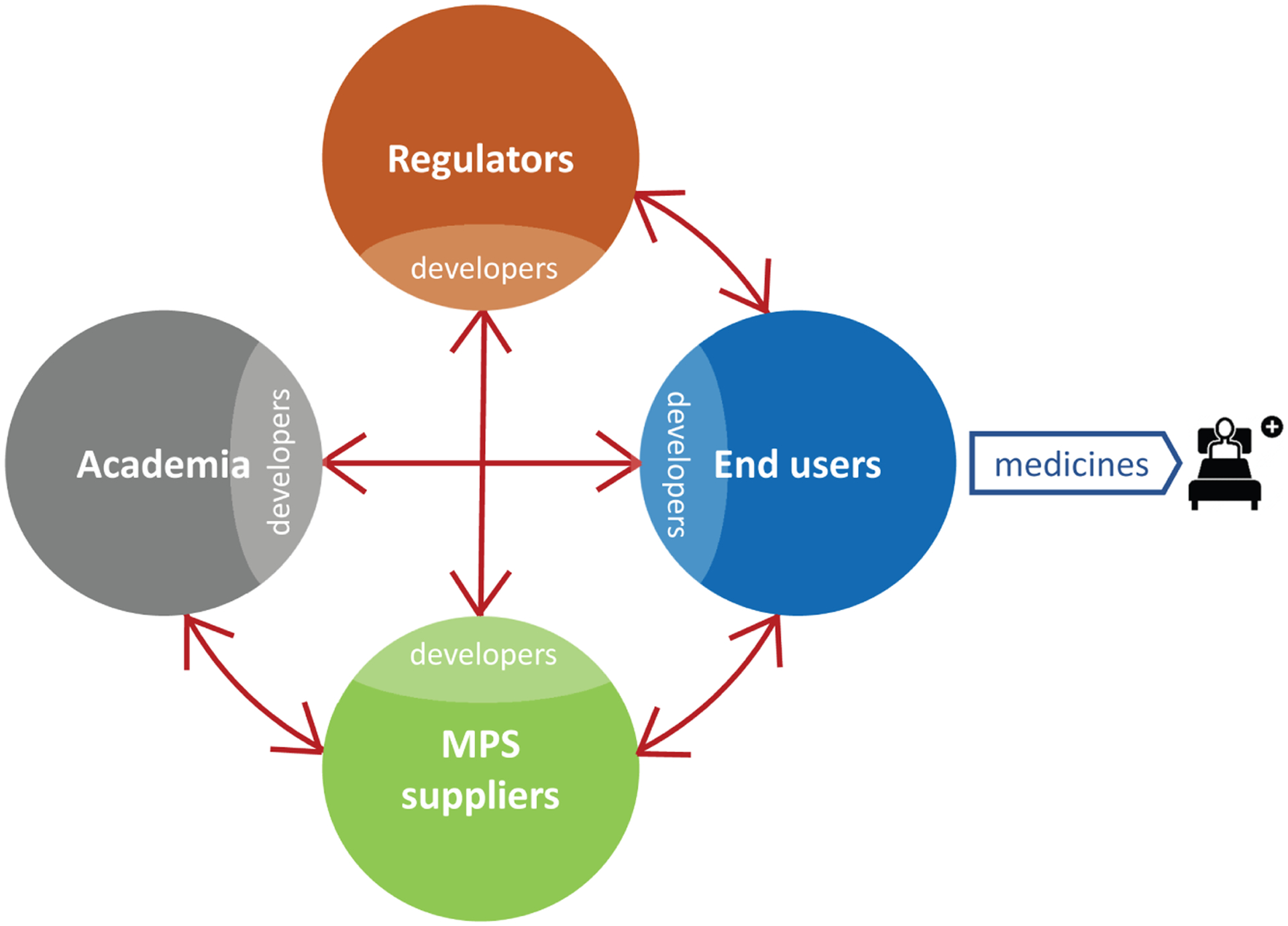
Smart stakeholder communication scheme

A smart, sustainable communication balance between the four stakeholders, schematically illustrated in Figure 8, is required to close the current communication gap on a global scale. To address this need, the workshop participants recommended the immediate actions summarized in Box 3. Proposed responsible entities for each activity are highlighted.

Workshop participants recommended the immediate actions summarized in Box 4 to support solving the qualification challenge for industrial adoption of MPS-based assays described in Section 5.

Workshop participants concluded that the immediate actions summarized in Box 5 could solve the regulatory acceptance dilemma for MPS-based assays described in Section 6 of this report.

Our recommendations should support the efficient establishment of ever more accepted MPS-based models and resulting MPS-based assays for specific contexts of use. We think one way is to focus developments primarily on assays serving new, cutting-edge medicines in areas of unmet medical needs lacking relevant animal models. Advanced therapies, such as CAR T-cell gene regenerative stem cell therapies are a prime example. Other areas to consider are the use of MPS for research in pregnancy, pediatrics and rare diseases or patient-derived MPS-based assays instead of patient-derived xenograft mice.

### 8.2 The roadmap

The experts at the workshop matched the recent developments in the field against the roadmap sketched at the first stakeholder workshop in June 2015 (Marx et al., 2016). They identified the pharmaceutical industry to become a first adopter of qualified MPS-based models and assays, with the chemical, cosmetics and food industries to follow. Therefore, here the authors focused on the impact of MPS systems on drug development.

**Fig. 9:**
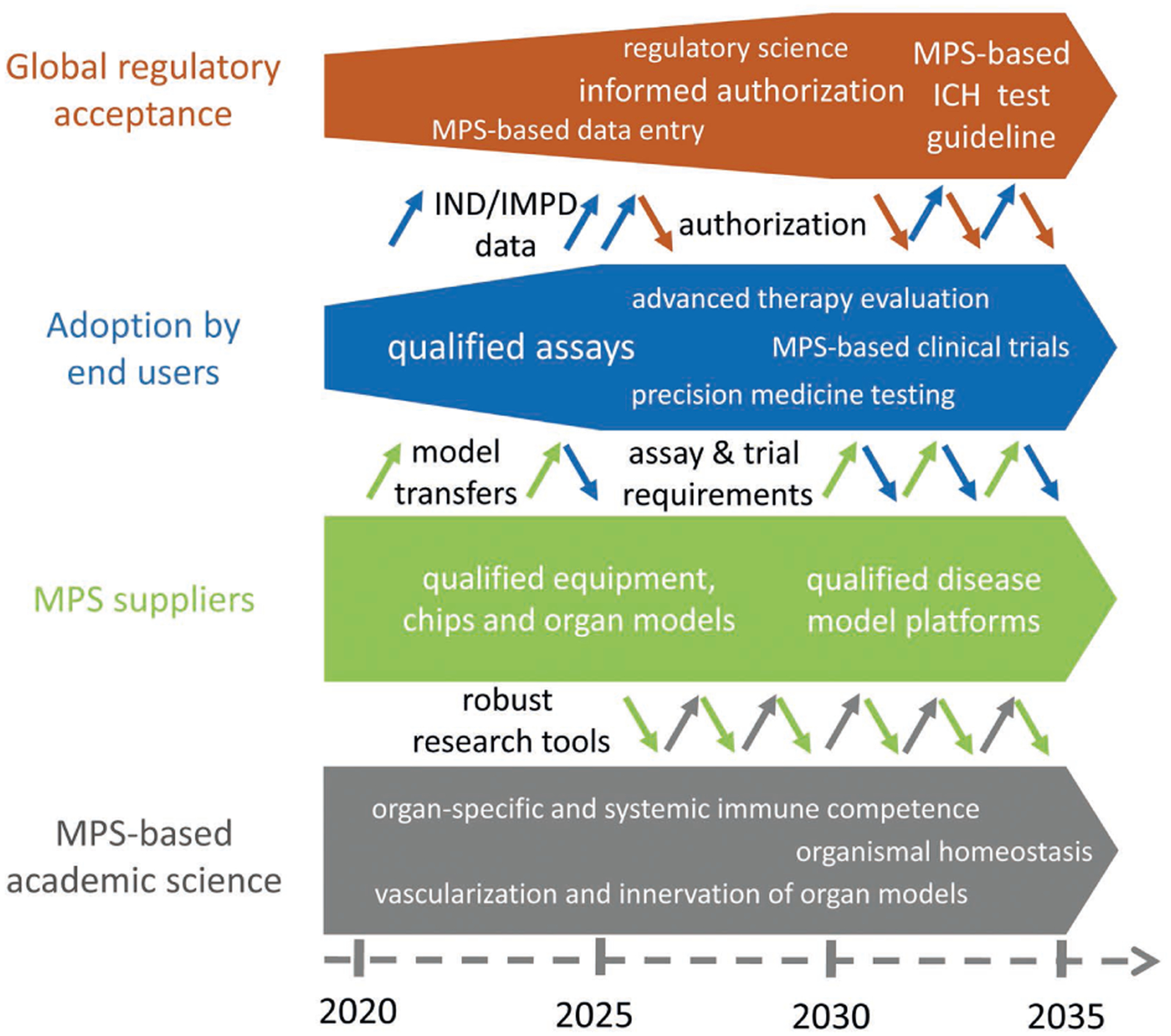
A roadmap toward patient benefit and animal welfare Brown, blue, green and grey arrows – influence of academia, MPS suppliers, end users and regulators, respectively, on other stakeholders in the process of development, transfer, use and data assessment of MPS-based models and assays.

Despite challenges and hurdles, early industrial adoption of first MPS-based assays by pharmaceutical industry has already become a reality (see Section 2.2). The roadmap has been updated on the basis of the workshop (Fig. 9). An ever-growing portfolio of qualified MPS-based assays fitting dedicated purposes will be adopted by the pharmaceutical industry for portfolio decision-making within the next five to ten years. MPS-based data for regulatory authorization of new drug candidates and advanced therapies will be entering IND and IMPD filings stepwise. Subsequently, within the next 15 years, we envision selected MPS-based assays to be validated to replace existing ICH guidelines on the use of laboratory animals where possible. In the chemical and cosmetics space, comparable processes towards alternatives to laboratory animal-based OECD guidelines are foreseen.

In order to achieve that goal, academia will continuously discover new and improve existing MPS-based models and methods to better emulate human biology. Integration of vasculature, innervation and systemic immunocompetence in MPS-based models are the major challenges in the next five to ten years, followed by organismal homeostasis models after 2030. Commercially valid models and methods from academia are taken up by the supplier industry, integrated into their commercial platforms, qualified according to end user needs and transferred to end users. Qualified disease models are envisioned to follow safety assays within five to ten years. Knowledge accumulated with model and assay qualification and widespread end user adoption will allow the supplier industry to feed robust MPS-based models and assays back into academia to be used for disruptive basic human research.

With growing trust and confidence in MPS at the end user level, the pharmaceutical industry’s range of purposes and their respective requirements and specifications to be met by the supplier industry will steadily increase (e.g., from healthy single-organ models toward disease models, from safety assays toward efficacy assays and on-chip clinical trial-like studies) within the next ten years. Data derived from MPS-based assays will reach regulatory recognition through IND and IMPD filing and a growing number of pre-submission case studies coordinated among the stakeholders. This, in turn, should increase regulatory awareness on a case-by-case and assay-by-assay basis and should stimulate the selection and validation of assay formats capable of replacing existing laboratory animal-based ICH guidelines 10 to 15 years from now.

The authors of this report are aware that this challenging roadmap, if successful, will not only lead to a paradigm shift in drug development and chemical safety assessment. However, it can only be successfully realized with all stakeholders equally involved.

### 8.3 Personalized organismal homeostasis on a chip remains a long-term vision

Human organismal MPS-based homeostasis was defined as the ultimate goal in emulating human biology to mimic mode of action and adverse outcome pathways (AOP) of therapies at a systemic level (Ingber and Whitesides, 2012; Marx et al., 2012; Shuler, 2012). It was envisaged that organs from at least the following ten systems of the human body should be interconnected to produce a minimal human organismal homeostasis *in vitro*: circulatory, endocrine, gastrointestinal, immune, integumentary, musculoskeletal, nervous, reproductive, respiratory and urinary (Marx et al., 2016). As of today, the three resulting approaches: i) the pumpless 14-compartment MPS of Michael L. Shulers group at Cornell University (Miller et al., 2016), ii) the physiome-on-a-chip of Linda G. Griffith’s group at MIT (Edington et al., 2018), and iii) the approach of Donald E. Ingber’s group at Wyss Institute to microfluidically link single-organ chips by an automated Interrogator instrument (Prantil-Baun et al., 2018; Herland et al., in press; Novak et al., 2020) have been applied to mimic this homeostasis *in vitro*. In contrast to the roadmap sketched in 2015 (Marx et al., 2016), such human-on-chip platforms cannot enter industrial adoption in 2020 due to their research development stage.

**Fig. 10:**
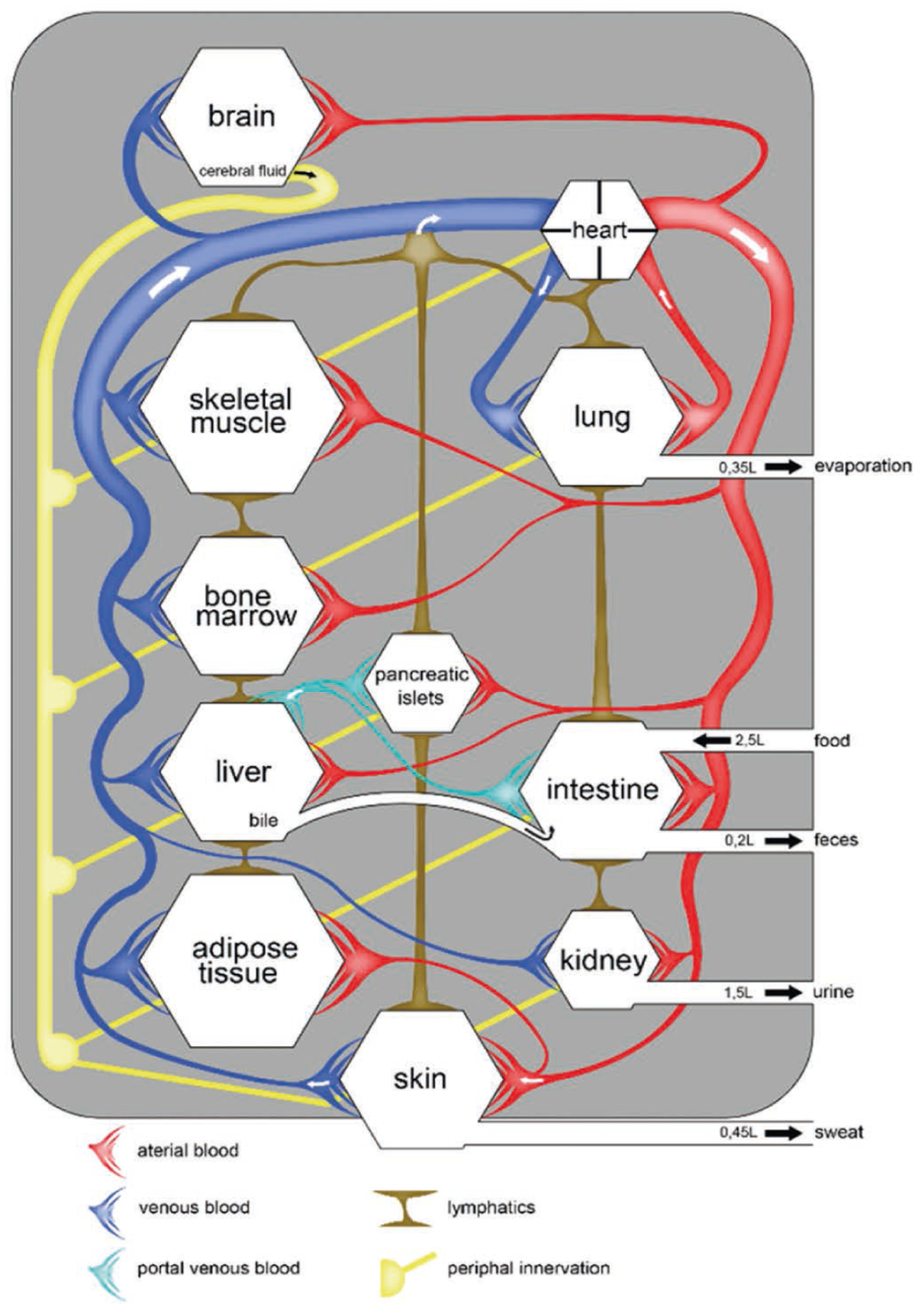
Schematic of a minimal set of human organs, their physiological connection through blood vessels and nerves, and the systemic physiological in- and output of a human to be downscaled to an MPS-based organismal model in order to create a universal physiological template (UPT)

In addition, the emphasis on how to approach systemic organismal homeostasis-on-chip has changed. The consensus of opinion has now turned towards establishing an MPS-based universal physiological template (UPT) incorporating self-sustained organismal homeostasis that can be used for basic physiological research and systemic disease modeling. Therefore, reaching the levels of biology sketched in the roadmap for MPS-based academic science is essential. The latter would need to fully emulate their respective functional units (e.g., architecture, blood perfusion, innervation, physiological turnover and stem cell niches) prior to template integration. Furthermore, integration should include respective interfaces and corresponding cross talk among functional units to enable, for example, hormone- or cytokine-driven homeostasis. A UPT should at least model the human organ combination illustrated in Figure 10 and meet the following process requirements:

- Air (oxygen) intake
- Food equivalent intake and processing
- Energy provision
- Whole blood production and provision
- Systemic regulation through innervation
- First line immune defense
- Excretion

A more detailed description of the organ equivalent systems involved and their functionality is explored in Table 5. External mimicry of hormonal environments (e.g., pituitary, pineal, thyroid, parathyroid and adrenal glands) by micro-formulator technologies is suggested instead of respective organ equivalent integration into UPT to mimic entire organismal functionality. The inclusion of the reproductive system could follow. Further development of UPT could also integrate additional factors, such as ethnic diversity based on autologous organ models and microbiomes.

**Fig. 11:**
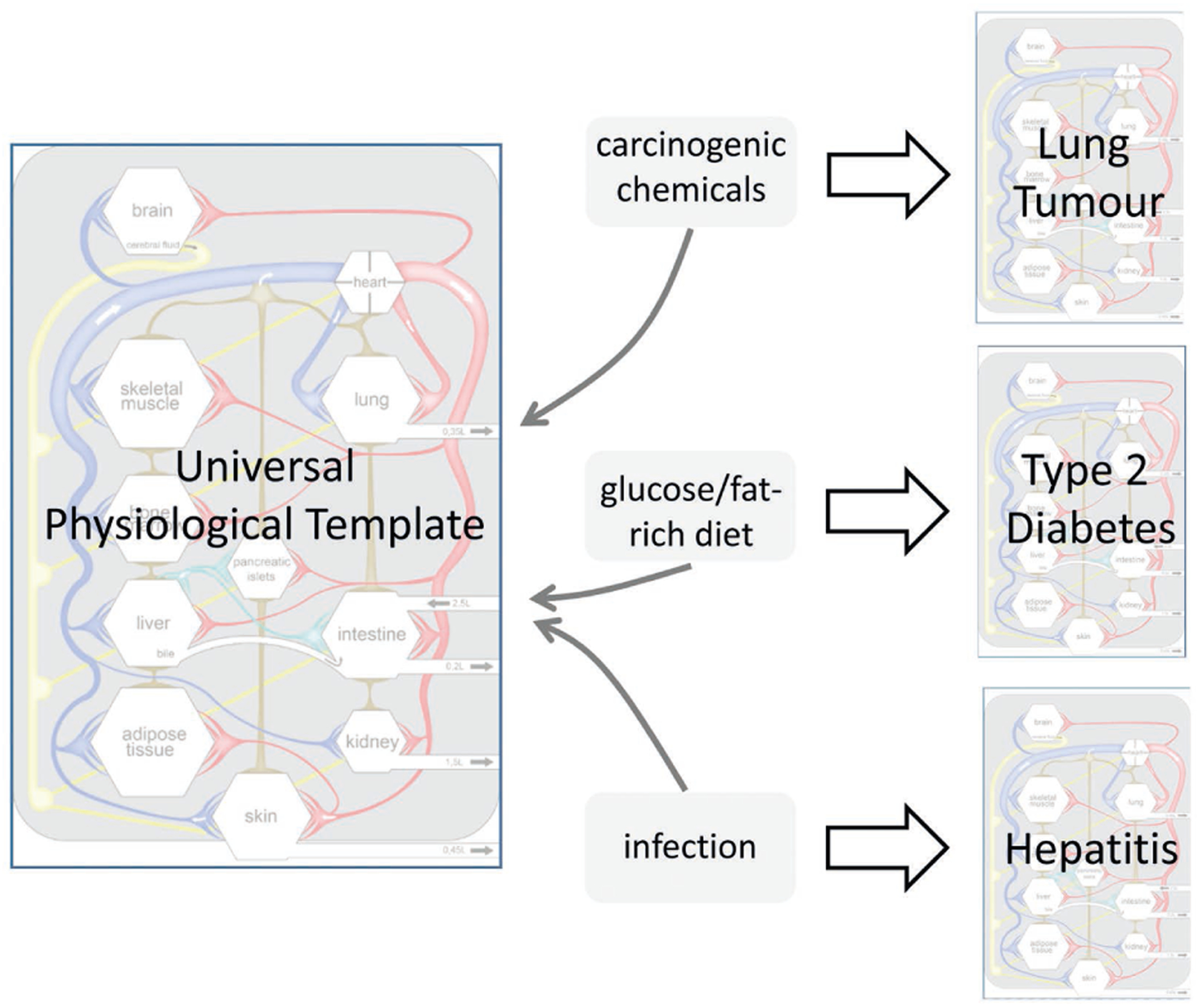
Schematic illustration of the creation of MPS-based disease modeling by treating a universal physiological template (UPT) with respective agents

Subsequently, the UPT can be developed into various disease models (Fig. 11). Therefore, naturally occurring mechanisms for the development of human pathophysiology could be explored to generate patient-on-chip platforms. Viral infection, exposure to carcinogenic chemicals for tumor induction or glucose and/or fat-rich diet for diabetes are prime examples. The resulting disease models can serve as basic research tools to investigate ethnic pathodiversity, human systemic pathogenesis or mode of action of advanced personalized therapies on a human organismal level. Moreover, large cohorts of personalized pathophysiological chips can form a basis for on-chip clinical trials (Beilmann et al., 2019) with the potential to disrupt the current drug development cycle.

Additional aspects to consider for the development of a UPT are the cell sources. Multiple cell sources are available, such as human cell lines, primary cells harvested from donors or patients, and stem cells (embryonic, fetal and iPSC) (Marx et al., 2016). The choice of the cell source should be based on the specific goal of the UPT being developed.

Finally, although having a UPT with self-sustained organismal homeostasis opens up wide opportunities to replace animal testing in basic research, foster personalized therapies and shift the drug development paradigm, ethical principles should be followed to define the scope of a study and its specific context. In addition, regulations are needed to oversee the use of such UPTs. Attention should be paid to the original cell donors’ right to rescind permission for subsequent use if necessary.

## 9 MPS for future patient benefit and laboratory animal welfare

The ethical dimension of human MPS technologies for our society is enormous. They bear the potential to significantly affect the health care systems and laboratory animal welfare of the future.

MPS will benefit patients on a large scale as soon as validated MPS-based context of use assays foster regulatory approval of advanced therapies and novel drugs. Furthermore, the application of such validated assays in the context of precision medicine selection using individual patient-derived tissues will make a difference for each of those patients.

Workshop participants envision that MPS platforms will reach the necessary qualification level within the upcoming decade. If they hold their promise to truly emulate human biology, MPS-based assays bear the potential to substantially increase a candidate’s success rate in clinical development (currently at 15%), which might result in an estimated twofold reduction of development time and fivefold reduction of drug development costs in the future (Marx et al., 2016). This, in turn, might have the largest impact on affordable pricing of novel therapies for any patient - nowadays another ethical dilemma in our society.

Furthermore, MPS will contribute to the reduction of laboratory animal testing in drug development as soon as a validated human MPS-based fit-for-purpose safety or efficacy assay enables informed cancellation of a portfolio candidate prior to its preclinical animal testing in the pharmaceutical industry. Authors could demonstrate that first early adopters are already or very soon will be in the position to do so.

In the long run, human MPS-based assays bear the potential to replace patient-derived xenograft animal models and to outperform newly developed humanized laboratory animal models recently introduced into advanced therapy testing.

Finally, MPS technologies are already competing intrinsically with laboratory animal models to satisfy humankind’s curiosity in basic and applied research. A large number of laboratories worldwide develop and use human MPS tools instead of implementing new CRISPR/Cas-derived genetically humanized animal models to understand human biology, invent new medicines or develop sustainable food.

**Fig. 12:**
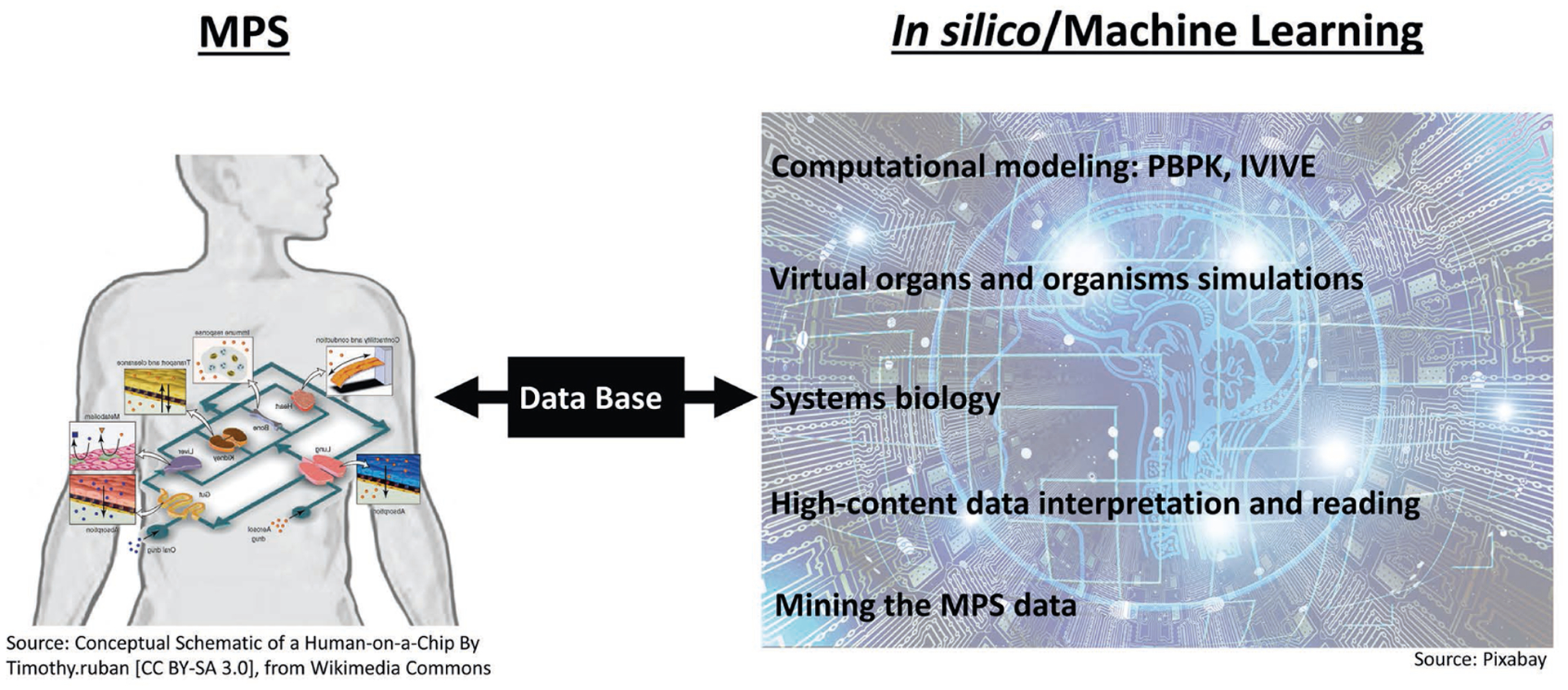
Schematic illustration of merging technologies of MPS and machine learning to continuously improve *in vitro* and *in silico* models towards the systemic organismal emulation of human (patho)biology Adopted from Smirnova et al. (2018).

With MPS technologies increasingly meeting artificial intelligence platforms at all levels of their life cycle (Fig. 12), training and feedback loops between the resulting human *in vitro* and *in silico* models are envisioned to move the field of disease modeling and safety and efficacy testing in drug development to a new level of bio-virtuality. Such developments will further increase the predictive value of preclinical data for patient benefit and decrease laboratory animal use in drug development.

All participants of the workshop collectively representing the four stakeholders in the field - academia, the MPS supplier industry, the end user industry (pharmaceutical industry, consumer industry, CROs) and regulatory bodies - are enthusiastic about contributing to the ethical impact on patient benefit and animal welfare in our society along the sketched roadmap.

## Figures and Tables

**Tab. 1:** MPS assays used for internal portfolio decision-making in drug development

MPS-based organ/tissue model	No. of cases	Area of use (drug development phase)	MPS-supplier	End user	Reference (if available)
Blood vessel, vasculature	5	Target identification, validation and compound selection	AIST	Daiichi-Sankyo	Satoh et al., 2016
		Discovery (scleroderma)	Mimetas	Galapagos	–
		Systems toxicology for consumer products	Mimetas	Philip Morris	Poussin et al.,2020
		Pharmacokinetics and pharmacology	Mimetas	undisclosed	–
		Target identification and validation	Mimetas	NovoNordisk	–
Bone marrow	4	Preclinical safety	TissUse	AstraZeneca	Sieber et al., 2018
		Preclinical safety	Emulate	AstraZeneca	Chou et al., 2018
		Preclinical safety	TissUse	Roche	–
		Preclinical safety	TissUse	Bayer	–
Gut epithelium	4	Discovery (inflammatory bowel disease)	Mimetas	Galapagos	Beaurivage et al., 2019
		Discovery	Mimetas	Roche	–
		Clinical development	Mimetas	Roche	–
		Preclinical safety	Emulate	Roche	–
Lung	3	Discovery (alveolus)	Wyss	undisclosed	Huh et al., 2012
		Drug efficacy (epithelium)	Wyss	Pfizer, Merck USA	Benam et al., 2016b
		Preclinical safety	Emulate	Roche	–
Liver	2	Pharmacological and toxicological effects	Emulate	AstraZeneca	Foster et al., 2019
		Preclinical safety - assessment of species (rat, dog & human)	Emulate	J&J, AstraZeneca	Jang et al., 2019
Ocular compartment	1	Discovery	Fh IGB / EKUT	Roche	Achberger et al., 2019
Kidney epithelium	1	Pharmacokinetics and pharmacology	Mimetas	undisclosed	Vormann et al., 2018
Liver-Pancreas	1	Target validation / identification	TissUse	AstraZeneca	Bauer et al., 2017
Liver-Thyroid	1	Preclinical safety – assessment of species-specificity (rat and human)	TissUse	Bayer	Kühnlenz et al., 2019
Skin-Tumor	1	Preclinical safety & efficacy	TissUse	Bayer	Hübner et al., 2019

Abbreviations: Wyss, Wyss Institute at Harvard, Boston, MA, USA; AIST, National Institute of Advanced Industrial Sciences, Tokyo, Japan; Fh IGB, Fraunhofer Institute for Interfacial Engineering and Biotechnology, Stuttgart, Germany; EKUT, Eberhard Karls University, Tübingen, Germany

**Tab. 2:** Quality assurance guidance for MPS-based assays

Component	Useful quality assurance guidance	Stakeholders responsible
MPS equipment including chips	Adhere to standard installation, operation and performance qualification (IQ, OQ, PQ) procedures. Different standards may cause irritation – need harmonization for critical parameters	MPS supplier assisted by developer
Cell culture conditions	Medium composition, growth factor ID, quality of documentation	Medium supplier assisted by developer
Cell sources	GCCP, GIVIMP, GTP, availability (avoid dependencies on single supplier), Define “fit-for-purpose” and “context-of-use” criteria for assay development, Harmonized conditions for primary cell preparation (e.g., culture medium, number of passages)	Cell supplier, e.g. cell bank assisted by developer, end user and regulators Cell supplier assisted by the developer
Organ or disease model	In-house qualification (reproducibility measures), Functionality assessment (e.g., TEER for skin models, CYP-cocktail testing)	Model supplier, end user, developer, academic labs^a^
Assay	(Guidance on) reference standard (if available), testing procedure (tools, dosages, endpoints), documentation, reproducibility	End user

a Academic labs are not covered by the term end user.

**Tab. 3:** Proposed end points and data of a shared MPS-based assay evaluation library

Parameter	Explanation
Compounds	Commercially available compounds
Context of use	A clear definition of the relevance of the test method, where relevance describes the relationship of the test method to the effect of interest and whether it is meaningful and useful for a particular purpose (https://www.ema.europa.eu/en/documents/scientific-guideline/guideline-principles-regulatory-acceptance-3rs-replacement-reduction-refinement-testing-approaches_en.pdf). This includes the endpoints which are to be investigated with reference to the conventional animal or human endpoint, e.g. if the MPS is used to detect DILI, it needs to be specified whether it covers all kinds of liver damage (cholestasis, steatosis, inflammation, fibrosis, …) and how these are specified (biomarkers, morphology, histopathology, …).
Historical reference data	Data describing morphological and physiological outcome (e.g., histopathology, clinical chemistry) in MPS for defined reference compounds (positive and negative controls). Concentration ranges tested should be included. Endpoints measured in the MPS might include genomics markers, biomarker changes, etc.
Cell material & quality	Description of cell or tissue source, including potential quality checks (e.g., viability, functional performance tests, metabolic activity)
Specification of materials & media	Detailed description of materials with regard to biocompatibility, potential leachables, surface adsorption (drug binding), composition of media (protein content and source, growth factors included in the medium or added, flow rates, etc.)
Exposure	Drug stability data and determination of exposure (total/unbound, ideally also intracellular)
Exposure modeling	Description of the model that was used to compare exposure in the MPS with the *in vivo* situation (animal or human)
General documentation	Will Good Laboratory Practice (GLP) need to be met for such studies? A workshop on this topic, perhaps in partnership with the OECD, would be advisable as it will inform any decisions on performance standards. Alternatively, the regulatory agencies could brainstorm on what context of use situation would require these systems to be performed under GLP.
Robustness	Intra-assay (repeatability) and inter-laboratory comparative result data. The need for the latter may be decided on a case-by-case basis.

**Tab. 4:** Challenges regulatory agencies are currently facing in the context of using MPS-based assay data for risk assessment and solutions to overcome hurdles

Challenges	Solutions
New technology lacking in-house experience regarding the evaluation and assessment of the MPS data acquired	Develop performance standards, acquire in-house experience via external training or in-house experimental setup
Time lag between use and first appearance of data at regulatory agencies	Encourage industry to submit compiled data outside a clinical trial or a marketing authorization application in order to characterize MPS-based assays. There is, for example, an evaluation of data outside regulatory decision-making as described in the *Guideline on the principles of regulatory acceptance of 3Rs testing approaches* (EMA, 2016)
Cultural reluctance to the adoption of new approaches	Strive for global harmonization regarding the use and assessment of MPS-based assay data
	Hold workshops regarding MPS-based assay data use and assessment in a regulatory context
	Collect and disseminate survey data on the use of MPS-based assays in the regulatory context
	Define context-of-use jointly with applicant and/or technology provider
	Define unmet needs of current regulatory approach (e.g., lacking concordance of animal study data with human outcome) as a first step towards regulatory acceptance

**Tab. 5:** Minimal set of organ models enabling respective functions to establish MPS-based organismal homeostasis

No.	System	MPS-based organ model	Function
1	Circulatory	Cardiovascular	Blood transport
		Bone marrow	Hematopoiesis
2	Nervous	Brain	Generating and processing neuronal signals
		Ganglions and nerves	Innervation
3	Immune	Innate immunity	First line defense
4	Respiratory	Lung	Blood oxygenation
5	Digestive	Intestine	Absorption barrier, hosting microbiome
		Liver	Metabolism, protein production
6	Urinary	Kidney	Excretion
7	Endocrine	Pancreatic islets	Glucose regulation via insulin
8	Integumentary	Skin	Barrier
		Adipose	Storage, signaling
9	Musculoskeletal	Muscle	Metabolic homeostasis
10	Reproductive (optional)	E.g., testis	Generation of sperm

## Box 1: The IQ Consortium

The IQ Consortium MPS Affiliate provides a unified voice from the pharma industry in working with NIH, FDA and tissue chip developers towards the implementation and qualification of MPS models as *in vitro* tools for drug development. As of October 2019, the following 22 companies are members of the affiliate: AbbVie, Amgen, Astellas, AstraZeneca, Biogen, Bristol Myers Squib, Celgene, Eisai, Eli Lilly, Genentech, GlaxoSmithKline, Jansen, Merck, Merck KgA, Mitsubishi Tanabe, Novartis, Pfizer, Sanofi, Seattle Genetics, Takeda, Theravance and Vertex.

## Box 2: Netherlands Organ-on-Chip Consortium hDMT

The role of hDMT is to develop and qualify cell culture models of healthy and diseased human tissues based on organ-on-chip technology and to facilitate valorization, implementation and availability of these models to end users tailored to their needs. The consortium consists of the following academic and industry partners: Amsterdam University Medical Center, Delft University of Technology, Eindhoven University of Technology, Erasmus University Medical Center, Genmab BV, Hubrecht Institute, Leiden University, Leiden University Medical Center, Maastricht University Medical Center, Radboud University Medical Center, TNO, University of Groningen, University of Twente, University Medical Center Groningen and Wageningen University and Research Institute. For detailed information, see www.hDMT.technology.

## Box 3: Closing the stakeholder communication gap

### EUROoCS and Japanese AMED leadership:

To found an international MPS society representing stakeholder activities in Europe, Asia and other parts of the world building on existing local society structures in the field. Major goals of the society are proposed to be the following:

- Organization of a biannual world congress on biology-inspired MPS providing a communication platform for all stakeholders under the leadership of EUROoCS (with rotating locations),
- Integration of current existing local Organ-on-Chip Conference and meeting formats into the world congress on MPS to reduce the total number of meetings and to significantly increase scientific quality,
- Involvement of new stakeholders such as patient groups as soon as MPS platforms have matured to serving their needs,
- Active development and supervision of global ethical standards for use of MPS-based technologies emulating human biology,
- Coordinated information of policy-makers and guidance of governmental, NGO-based and philanthropic funding programs (e.g., DARPA, IMI 2, NC3R, Bill & Melinda Gates and Mark Zuckerberg foundations) regarding funding bottlenecks in the field, and
- Establishment of sustainable workshops and training programs for young scientists and technicians in the field, across the globe.

### Academia:

To support open access journals solely focussing on basic and applied science discoveries with microfluidic MPS such as the new Elsevier journal “Organs-on-a-Chip”.

### CAAT Europe:

To repeat the CAAT stakeholder workshop on biology-inspired MPS on a three-year basis.

## Box 4: How to support MPS-based assay qualification activities

### All stakeholders:

To foster the further growth and establishment of centers for the evaluation and validation of MPS-based methods in America, Europe and Asia under the supervision of regulators. Involve all stakeholders in co-funding of such centers and ensure coordination of the evaluation and validation programs of such centers to avoid redundancy across the globe.

### End users (Pharma, CROs):

To become members of the IQ consortium and actively pursue MPS-based assay qualification strategies there.

### End users (Pharma, Biotech):

To jointly fund or support existing funding for MPS-based assay qualification in various settings including centers of excellence (virtual CRO’s are also to be considered) to centrally assess MPS-based assays for a given context of use. This should lower the hurdle for late adopters of MPS-based assays in big pharmaceutical industry and for smaller pharmaceutical companies and biotechs.

## Box 5: Addressing the regulatory acceptance dilemma

### End users and suppliers:

To make internal feasibility studies on MPS-based tests with a given fit-for-purpose publicly available, wherever possible.

### End users, suppliers and regulators:

To generate use cases for MPS-based assays under the supervision of an end user.

### Regulators:

To produce a position paper on aspects of regulatory acceptance of biology-inspired MPS-based assays for drug testing under supervision of FDA involving American, European and Asian regulatory agencies. This should guide sponsors to learn how the agencies deal with the data based on case examples.

### Regulators:

To establish a standardized annual meeting format to convene a group of regulators from America, Europe, Asia and other geographies in the drug, food, and biologics space

- coordinating regulatory science,
- tracking and analyzing MPS-based data arrival at a regulatory level (e.g., IMPD, IND),
- organizing the development of ICH guidelines for MPS-based assays that make it to replace existing animal-based ICH guidelines.

## Footnotes

1 https://www.fda.gov/science-research/about-science-research-fda/fdas-predictive-toxicology-roadmap

2 https://ncats.nih.gov/tissuechip/projects/centers/2018 (accessed 12.11.2019)

3 https://ncats.nih.gov/tissuechip/projects/modeling (accessed 12.11.2019)

4 https://go-bio.de/en/multi-organ-bioreactor-chip-format (accessed 12.11.2019)

5 https://noci-organ-on-chip.nl/ (accessed 12.11.2019)

6 https://www.organonachip.org.uk/ (accessed 12.11.2019)

7 https://www.bodyonchip.fi/ (accessed 12.11.2019)

8 https://www.med.uio.no/hth/english/ (accessed 12.11.2019)

9 https://h2020-orchid.eu/ (accessed 12.11.2019)

10 https://www.euroocs.eu/ (accessed 12.11.2019)

11 https://www.eurooc.eu/ (accessed 12.11.2019)

12 https://ncats.nih.gov/tissuechip/projects/centers/2016 (accessed 12.11.2019)

13 https://ncats.nih.gov/tissuechip (accessed 12.11.2019)

14 Microphysiology Systems Database. University of Pittsburgh Drug Discovery Institute. https://mps.csb.pitt.edu/ (accessed 12.11.2019)

15 http://www.scetra.or.jp/en/ (accessed 12.11.2019)

16 https://www.fda.gov/science-research/about-science-research-fda/emerging-sciences (accessed 12.11.2019)

17 https://www.fda.gov/science-research/about-science-research-fda/fda-science-forum (accessed 12.11.2019)

## Abbreviations

CRO: contract research organization
DARPA: US Defense Advanced Research Projects Agency
EMA: European Medicines Agency
EPA: US Environmental Protection Agency
FDA: US Food and Drug Administration
ICH: International Council for Harmonisation of Technical Requirements for Pharmaceuticals for Human Use
IMPD: investigational medicinal product dossier
IND: investigational new drug
iPSC: induced pluripotent stem cell
MIT: Massachusetts Institute of Technology
MPS: microphysiological system
NCATS: National Center for Advancing Translational Sciences
NIFDC: National Institutes for Food and Drug Control
NIH: US National Institutes of Health
OECD: Organisation for Economic Co-operation and Development
SOP: standard operating procedure
TCTC: Tissue Chip Testing Center
UPT: universal physiological template
